# Age-dependent gray matter demyelination is associated with leptomeningeal neutrophil accumulation

**DOI:** 10.1172/jci.insight.158144

**Published:** 2022-05-10

**Authors:** Michelle Zuo, Naomi M. Fettig, Louis-Philippe Bernier, Elisabeth Pössnecker, Shoshana Spring, Annie Pu, Xianjie I. Ma, Dennis S.W. Lee, Lesley A. Ward, Anshu Sharma, Jens Kuhle, John G. Sled, Anne-Katrin Pröbstel, Brian A. MacVicar, Lisa C. Osborne, Jennifer L. Gommerman, Valeria Ramaglia

**Affiliations:** 1 Department of Immunology, University of Toronto, Toronto, Ontario, Canada.; 2 Department of Microbiology and Immunology and Life Sciences Institute, and; 3 Department of Psychiatry, University of British Columbia, Vancouver, British Columbia, Canada.; 4 Multiple Sclerosis Center & Research Center for Clinical Neuroimmunology and Neuroscience Basel (RC2NB), Departments of Neurology, Biomedicine, and Clinical Research, University Hospital and University of Basel, Basel, Switzerland.; 5 Mouse Imaging Centre, Hospital for Sick Children, Toronto, Ontario, Canada.; 6 Department of Medical Biophysics, University of Toronto, Toronto, Ontario, Canada.

**Keywords:** Immunology, Multiple sclerosis

## Abstract

People living with multiple sclerosis (MS) experience episodic CNS white matter lesions instigated by autoreactive T cells. With age, patients with MS show evidence of gray matter demyelination and experience devastating nonremitting symptomology. What drives progression is unclear and studying this has been hampered by the lack of suitable animal models. Here, we show that passive experimental autoimmune encephalomyelitis (EAE) induced by an adoptive transfer of young Th17 cells induced a nonremitting clinical phenotype that was associated with persistent leptomeningeal inflammation and cortical pathology in old, but not young, SJL/J mice. Although the quantity and quality of T cells did not differ in the brains of old versus young EAE mice, an increase in neutrophils and a decrease in B cells were observed in the brains of old mice. Neutrophils were also found in the leptomeninges of a subset of progressive MS patient brains that showed evidence of leptomeningeal inflammation and subpial cortical demyelination. Taken together, our data show that while Th17 cells initiate CNS inflammation, subsequent clinical symptoms and gray matter pathology are dictated by age and associated with other immune cells, such as neutrophils.

## Graphical abstract

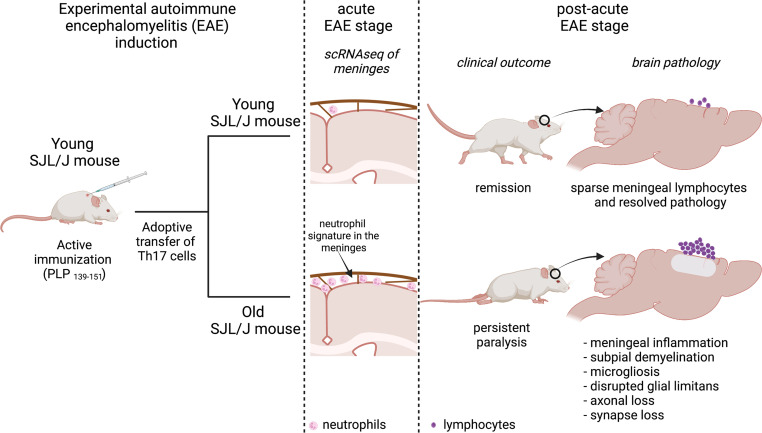

## Introduction

Multiple sclerosis (MS) is an autoimmune disease that causes demyelination of the CNS. MS is typically diagnosed in early adulthood as relapsing-remitting MS (RRMS) (1). Disease activity waxes and wanes, with relapses characterized by lesion formation in the deep white matter initiated by infiltration of autoreactive T lymphocytes across the blood-brain barrier (2). Approximately 15–20 years following onset, or when symptoms are first manifested in people older than 40 years, the disease enters a progressive phase, becoming nonremitting (3). Age is a major risk factor for disease progression in MS (4), with epidemiological studies showing that older chronological age at onset is associated with faster time to disability milestones (3, 5–7).

A key hallmark of brain pathology in progressive MS is demyelination and neurodegeneration of the gray matter. Although present from the earliest stages of MS (8, 9), gray matter injury accrues with disease progression (10) and associates with motor deficits and cognitive impairments (11, 12), and extensive cortical damage at onset predisposes to a rapid transition into the progressive phase of the disease (13). Unfortunately, the process of disease progression is ill understood. Notably, immunomodulatory therapies that are effective at suppressing relapsing disease have at best little impact on progression (14).

It has been proposed that immunomodulatory therapies fail in progressive MS (PMS) because disease progression is not governed by immune cells (15) despite immune cells being found in brain-adjacent regions of the PMS brain, in particular within the leptomeninges (16–18). Moreover, these immune cells appear to be proximal to areas of gray matter injury (19–21) and patients with aggregates of leptomeningeal immune cells harbor a number of proinflammatory molecules in their cerebrospinal fluid (CSF) (22). Thus, it is likely that immune cells are involved in the clinical and pathological presentation of PMS, but therapies that are used in RRMS do not target these immune cells, or there are redundant immune cell types in the leptomeninges that contribute to cortical pathology that cannot be erased by a singular immunomodulatory drug.

To gain mechanistic insights into what drives disease progression in aging patients with MS, an animal model that replicates nonremitting clinical disability that is accompanied by unrelenting gray matter demyelination and neurodegeneration is required. We have previously shown that experimental autoimmune encephalomyelitis (EAE) induced by adoptive transfer of encephalitogenic Th17 cells into young (6 weeks old) SJL/J recipient mice results in stromal cell remodeling within the brain leptomeninges that is accompanied by chemokine and cytokine expression. This stromal cell remodeling creates an immunocompetent niche in the leptomeninges (23) that is spatially associated with subpial cortical gray matter demyelination, microglial/macrophage activation, disruption of the glial limitans, and evidence of an oxidative stress response (24). However, there was no evidence of synapse loss or axonal damage in these mice, and gray matter pathology was transient, with mice recovering from the initial inflammatory event (24).

In the present study, we tested the effect of age on the clinical outcome of adoptive/transfer (A/T) EAE by transferring young encephalitogenic Th17 cells into young (6 weeks old) versus old (8 to 15 months old) SJL/J recipient mice. The age of the recipient mice had a profound impact on clinical phenotype and brain pathology. Whereas young recipients underwent disease remission, signs of paralysis were more severe and sustained in old mice. Old mice exhibited numerous and large aggregates of immune cells in the leptomeninges overlying regions of cortical injury and brain atrophy. Single-cell RNA sequencing of leptomeningeal resident cells identified a number of gene expression changes that are unique to the aged EAE phenotype, which led us to examine the differential abundance of neutrophils and B cells in the leptomeninges of old versus young mice. Importantly, we validated the presence of neutrophils in the brains of a subset of patients with PMS via postmortem human brains collected at rapid autopsy. Collectively, our study provides a model for studying Th17 cell–induced gray matter injury and sheds light on potential drivers of age-dependent MS disease progression.

## Results

### A/T of encephalitogenic Th17 cells into old SJL/J mice results in nonremitting EAE that is independent of vivarium and sex.

**Figure 1:**
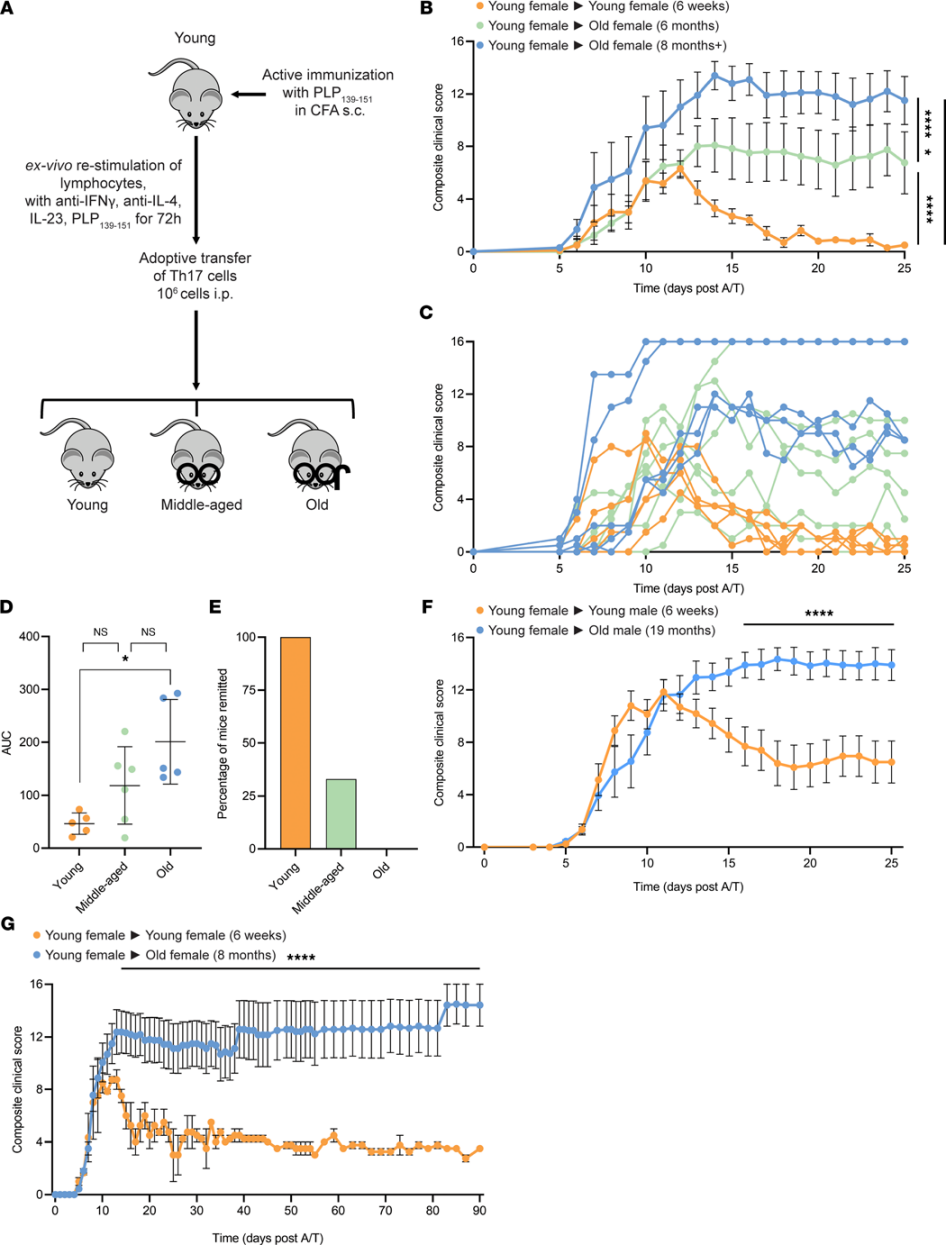
Aging determines clinical course in SJL/J A/T EAE mice, which is independent of sex and vivarium and persists over multiple months. (**A**) Induction of A/T EAE in SJL/J mice. (**B**) Average and (**C**) individual composite clinical scores (16-point scale) of young (6 weeks, *n* = 5), middle-aged (6 months, *n* = 6), and old (8 months, *n* = 5) female A/T SJL/J EAE mice receiving cells from young (6 weeks) female donors primed with PLP_139-151_. (**D**) AUC of individual mice. Statistical analysis performed by 1-way ANOVA with Bonferroni correction for multiple comparisons; error bars indicate mean ± SD. (**E**) Percentage of mice remitted in each group. (**F**) Composite clinical score of young (*n* = 10) and old (*n* = 10) male A/T SJL/J EAE mice receiving cells from young female donors primed with PLP_139-151_. (**G**) Composite clinical score of young (*n* = 2) and old (*n* = 8) female SJL/J A/T EAE mice followed for up to 90 days after A/T. (**B**, **F**, and **G**) Statistical analysis by 2-way ANOVA with Bonferroni’s correction for multiple comparisons; error bars indicate mean ± SEM. Experiments in **B** and **C** were performed at UBC whereas experiments in **F** and **G** were performed at U of T. *P ≤ 0.05, ****P ≤ 0.0001.

We have previously shown that A/T of encephalitogenic Th17 cells into SJL/J recipient mice induces EAE, with clinical symptoms first observed at approximately 5 days post-A/T, peaking at approximately days 11–12 (23, 24), and recovering shortly thereafter around day 14 (Figure 1A). An important advantage of using the A/T EAE model is that the T cell priming occurs in the donor mouse. Therefore, analyzing EAE in recipient mice allows us to focus on the effector phase of the disease, in isolation from the priming phase and away from the potential adjuvant-related effects that could confound interpretation of the data. Since age is the strongest predictor of progression (21, 25), we tested whether transfer of young Th17 cells into old recipient mice would alter the clinical course of EAE (Figure 1A). We found that old female mice (8 months) that received proteolipid protein–primed (PLP-primed) Th17 cells from young mice failed to remit, sustaining disability with average clinical scores of 11–13. Middle-aged mice (6 months) displayed an intermediate phenotype, with some mice experiencing remission and others exhibiting nonremitting disease, suggesting that 6 months of age is an inflection point between remission and nonremission phenotypes (Figure 1, B and C). We observed an increase in severity of disease with increasing age (Figure 1D) and a decrease in rate of remission (Figure 1E) in old SJL/J A/T EAE mice (0% remission) versus middle-aged (33% remission) versus young mice (100% remission). Disease in both young and old mice was specific to encephalitogenic PLP_139-151_–primed T cells since transfer of cells from donors immunized with OVA_326-339_ failed to induce EAE (Supplemental Figure 1A; supplemental material available online with this article; https://doi.org/10.1172/jci.insight.158144DS1). We also confirmed that the old versus young phenotype could be replicated in different vivaria (University of British Columbia [UBC] and University of Toronto [U of T]) (Figure 1, B, F, and G), suggesting that age — not housing conditions — is the main driver of this clinical phenotype. Last, we found that the nonremitting disease course was reproduced in older mice up to 15 months of age, with survival rate decreasing with increasing age (Supplemental Figure 1); was reproduced in male recipient mice (Figure 1F); and persisted over several months (Figure 1G).

### A/T EAE in old SJL/J mice results in persistent leptomeningeal inflammation and gray matter pathology.

**Figure 2:**
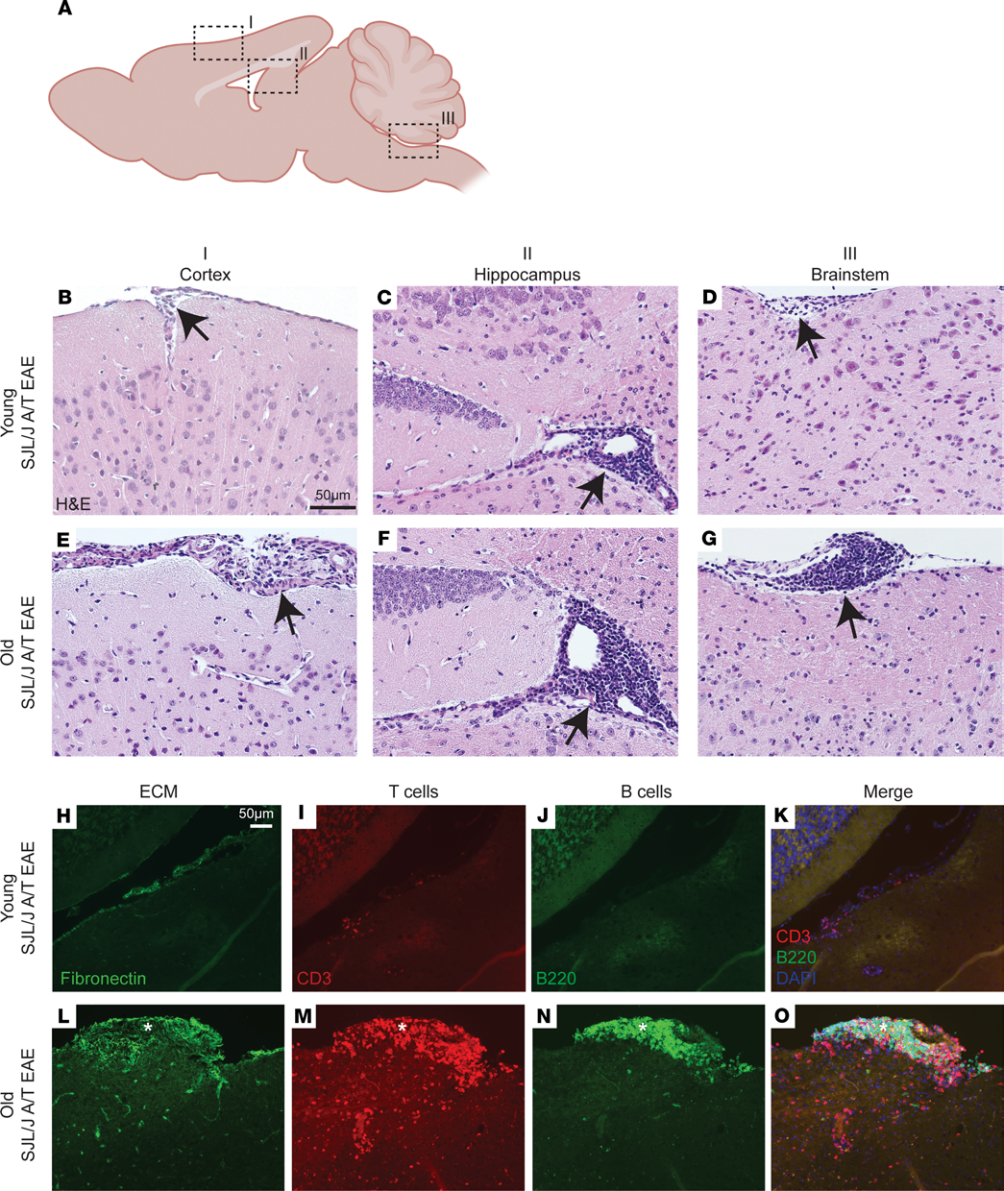
Aging induces accumulation of lymphocytes in leptomeninges adjacent to subpial and periventricular areas in SJL/J A/T EAE mice at postacute disease stage. (**A**) Schematic of mouse brain with the sagittal plane in view showing (I) cortex, (II) hippocampus and lateral ventricle, and (III) brainstem and fourth ventricle regions. Representative sagittal sections of brains obtained from (**B**–**D**) young versus (**E**–**G**) old SJL/J A/T EAE mice at day 25 after adoptive transfer. Brains of old mice show infiltration of cells in the leptomeninges in all 3 locations denoted by black arrows. (**H** and **L**) Immunofluorescence staining for fibronectin (ECM) revealing an elaborate fibronectin^+^ ECM network (denoted with *). Staining of serial sections for (**I** and **M**) CD3 and (**J** and **N**) B220 reveals CD3^+^ T cells and B220^+^ B cells present within this fibronectin+ meningeal niche (**K** and **O**). Scale bars: 50 μm.

We have previously shown that A/T of encephalitogenic Th17 cells into young SJL/J recipient mice provokes the formation of leptomeningeal immune cell aggregates overlying areas of subpial demyelination at the acute phase of disease, particularly in the proximity of the cortex, hippocampal fissure, and brainstem (Figure 2A) (23). Histological analysis of brain tissue at day 25 post-A/T, a time point when young mice have largely remitted in terms of their clinical scores, revealed a trend toward larger and more numerous aggregates of immune infiltrates in the brain leptomeninges in old recipients compared with young recipients (Figure 2, B–G). This was especially apparent in the leptomeninges proximal to the brainstem (Supplemental Figure 2, A and B). Quantification of imaging data in cortex, hippocampus, and brainstem regions revealed a trend toward increased numbers of leptomeningeal aggregates and a significant increase in aggregate area in the hippocampus (mean difference = 0.026 mm^2^/tertiary lymphoid tissue [TLT]) and brainstem (mean difference = 0.029 mm^2^/TLT) (Supplemental Figure 2, A and B) in old versus young A/T EAE mice. Further examination of these aggregates by immunofluorescence (IF) revealed that, consistent with our previous observations (24), leptomeningeal aggregates contained CD3^+^ T cells and B220^+^ B cells (Figure 2, I–K and M–O) and were associated with a network of fibronectin^+^ extracellular matrix (Figure 2, H and L), and histological scoring of these aggregates revealed an increase in numbers of B220^+^ B cells and CD3^+^ T cells in the cortex, hippocampus, and brainstem of old mice (Supplemental Figure 2, C and D). These data provide evidence that these leptomeningeal aggregates, which were greater in number and area in old SJL/J A/T EAE mice, were composed of CD3^+^ T cells and B220^+^ B cells.

**Figure 3:**
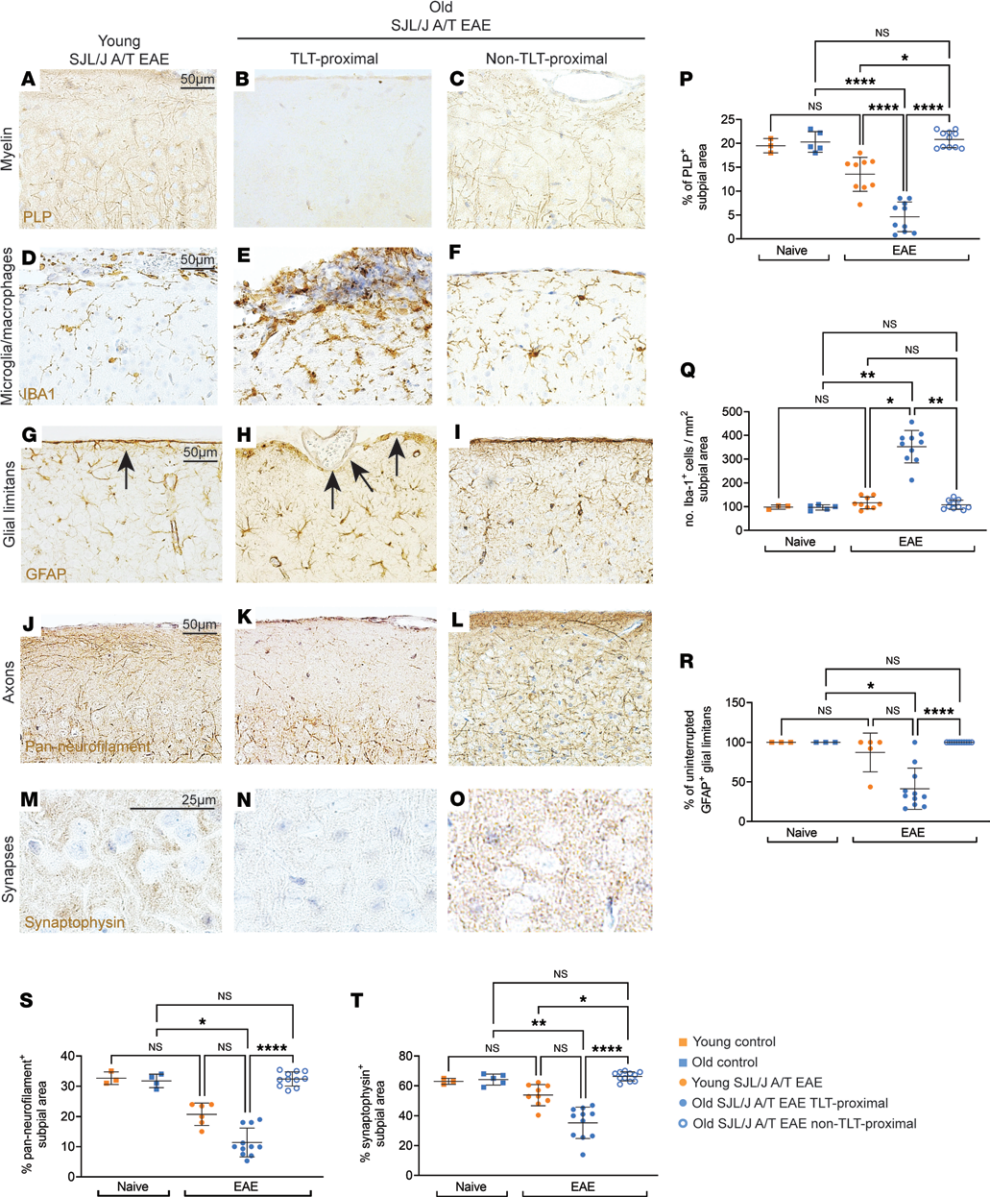
Sustained subpial cortical injury is found in close proximity to regions of leptomeningeal infiltration in old SJL/J A/T EAE mice. Assessment of the subpial somatosensory cortex of young (*n* = 6) versus old (*n* = 11) SJL/J A/T EAE mice at the postacute stage (day 25 after A/T) by immunohistochemistry (IHC) in TLT-proximal (filled circles) versus non-TLT-proximal regions (open circles). Controls indicated are unimmunized, age-matched mice (*n* = 3–5 in each group). (**A**–**C** and **P**) Myelin content (PLP); (**D**–**F** and **Q**) Macrophage/microglia density (Iba1); (**G**–**I** and **R**) glial limitans and astrocytes (GFAP), black arrows = glial limitans; (**J**–**L** and **S**) axons (pan-neurofilament); (**M**–**O** and **T**) synapses (synaptophysin). (**P**, **Q**, **S**, and **T**) Statistical analysis by 1-way ANOVA with Tukey’s correction for multiple comparisons. (**R**) Statistical analysis by Kruskal-Wallis with Dunn’s correction for multiple comparisons. Error bars indicate mean ± SD. *P ≤ 0.05, **P ≤ 0.01, ****P ≤ 0.0001. Scale bars: 50 μm except for 25 μm (**M**–**O**).

To ascertain the impact of SJL/J A/T EAE on the gray matter in old versus young recipient mice, we performed immunohistochemistry (IHC) for PLP (myelin), glial fibrillary acidic protein (GFAP, astrocytes), ionized calcium-binding adapter molecule 1 (Iba1, microglia/macrophages), neurofilament (axons), and synaptophysin (synapses). At day 25 post A/T, while we did not observe gray matter injury proximal to non-TLT-proximal areas, in brain regions adjacent to the leptomeningeal TLT, we observed a significant increase in demyelinated subpial area (Figure 3, A–C and P), and density of microglia/macrophages (Figure 3, D–F and Q), as well as a trend toward enhanced disruption of the glial limitans (Figure 3, G–I and R), decreased axonal integrity (Figure 3, J–L and S), and decreased synaptophysin^+^ area (Figure 3, M–O and T) in old versus young SJL/J A/T EAE mice. These data indicate that old mice exhibit enhanced gray matter injury compared with young mice at day 25 post-A/T in areas adjacent to TLTs.

**Figure 4:**
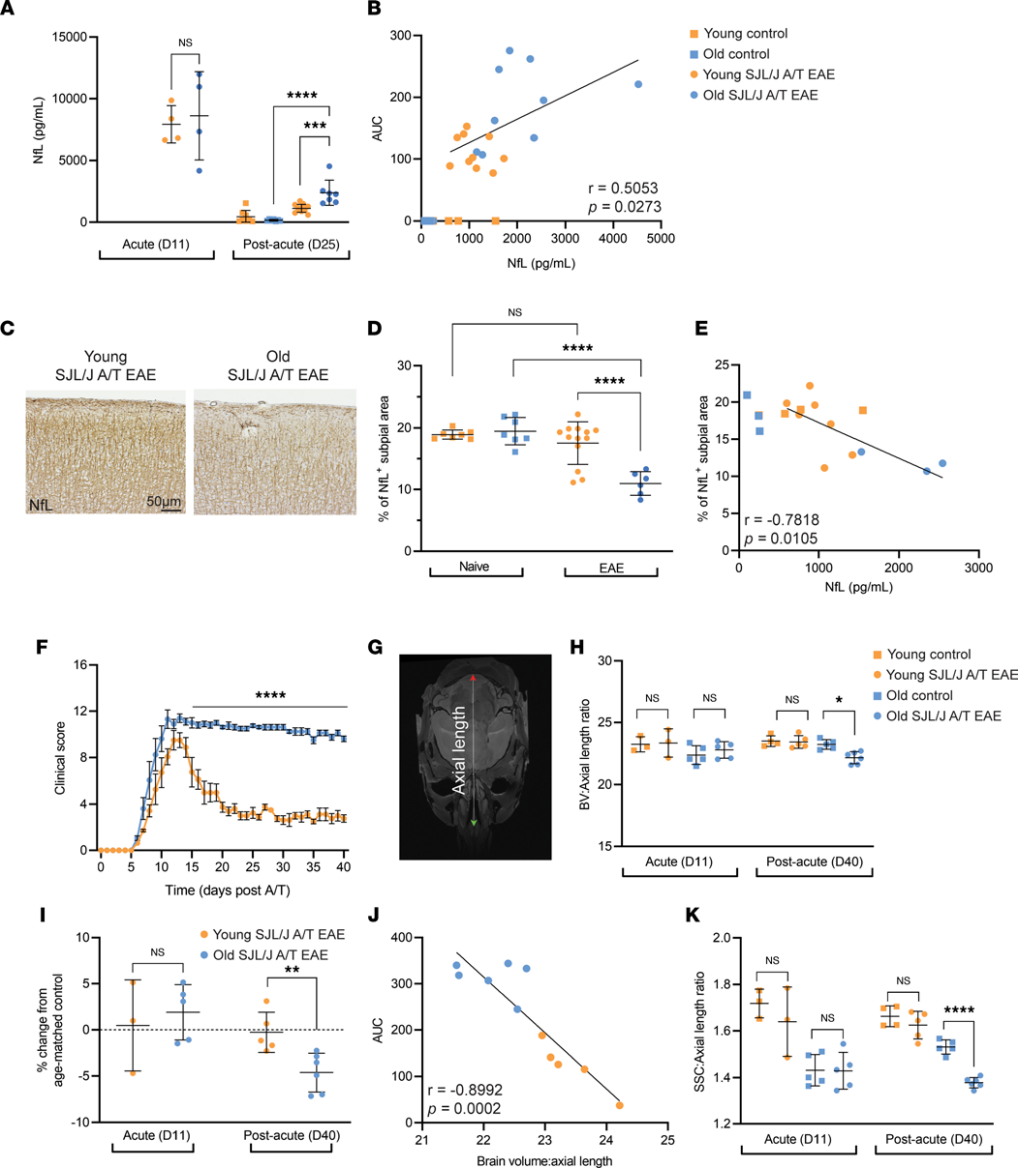
Aging induces accumulation of NfL in SJL/J A/T EAE mice and loss of brain volume at the postacute disease stage. (**A**) Serum neurofilament light chain (NfL) levels in young (*n* = 4–12) and old (*n* = 4–7) SJL/J A/T EAE mice at the acute (day 11) and postacute (day 25) disease stages. Age-matched, unimmunized controls included for comparison at the postacute time point (young, *n* = 4; old, *n* = 4). (**B**) Correlation analysis of clinical severity represented by AUC with levels of serum NfL. (**C**) Representative images in the somatosensory cortex (SSC) of old versus young SJL/J A/T EAE mice. (**D**) Quantification of IHC staining for NfL using %NfL^+^ area. (**E**) Correlation analysis of NfL immunohistochemistry and serum levels. (**F**) Clinical course of old (*n* = 8) versus young (*n* = 8) SJL/J A/T EAE mice followed for 40 days post-A/T. Statistical analysis by 2-way ANOVA with Bonferroni’s correction for multiple comparisons; error bars indicate mean ± SD. (**G**) Ex vivo MRI image of an SJL/J A/T EAE mouse showing the axial length of the skull from the nasal cavity (green arrow) to the base of the skull (red arrow). (**H**) Brain volume of young (*n* = 3–5) versus old (*n* = 5–6) SJL/J A/T EAE mice expressed as a ratio to axial length. (**I**) Percentage change in SJL/J A/T EAE mice from age-matched controls (young *n* = 3, old *n* = 3) at acute and postacute disease stages. Statistical analysis by Mann-Whitney *U* test; error bars indicate mean ± SD. (**J**) Correlation analysis of clinical severity with brain volume. (**K**) SSC volume of each mouse expressed as a ratio to axial length. (**A**, **H**, and **K**) Statistical analysis by 1-way ANOVA with Bonferroni’s correction for multiple comparisons; error bars indicate mean ± SD. (**D**) Statistical analysis by Kruskal-Wallis with correction for multiple comparisons; error bars indicate mean ± SD. (**B** and **E**) Statistical analysis by Spearman’s correlation test. (**J**) Statistical analysis by Pearson’s correlation test. *P ≤ 0.05, **P ≤ 0.01, ***P ≤ 0.001, ****P ≤ 0.0001.

Serum neurofilament light chain (NfL) has become an increasingly used biomarker for ascertaining MS severity (26). To test whether NfL is induced by A/T of Th17 cells, we subjected serum from old versus young SJL/J A/T EAE mice to Quanterix single-molecule array (Simoa) technology. At peak disease, old and young mice exhibited similar levels of serum NfL (Figure 4A). However, at the postacute phase of disease, old recipients exhibited augmented NfL compared with sex-matched young recipients (mean old = 2384 pg/mL, mean EAE young = 1123 pg/mL) (Figure 4A), and NfL levels positively correlated with disease severity (Spearman’s *r* = 0.5053, *P* = 0.0273) (Figure 4B). To determine whether NfL levels reflect ongoing neuronal damage in the subpial cortex, we assessed brain tissues from corresponding mice by IHC. Old mice exhibited a lower percentage of NfL^+^ area in the subpial cortex compared with young mice (mean EAE old = 11.0% NfL^+^ area, mean young = 17.5% NfL^+^ area, *P* < 0.0001) (Figure 4, C and D), and these values negatively correlated with NfL levels (Spearman’s *r* = –0.7818, *P* = 0.0105) (Figure 4E), suggesting that increased serum NfL levels reflected a decrease in NfL in the cortex. Therefore, NfL levels correlated with worse clinical outcomes in old mice, and elevated NfL in old mice was driven at least in part by neuronal damage in the cortex.

A key hallmark of progressive MS is a reduction in brain volume driven in part by atrophy in the cortical gray matter (27, 28). To test if the SJL/J A/T EAE model in old mice exhibits brain atrophy, we followed old and young SJL/J mice for 40 days post-A/T and assessed brain volume by T2-weighted 7-Tesla magnetic resonance imaging (MRI) at 2 time points — acute (day 11) and postacute (day 40) (Figure 4F). Normalizing to skull length measurements taken from the nasal cavity to the base of the skull (the axial MRI view — see Figure 4G), we found that old SJL/J A/T EAE mice exhibited lower brain volume compared with age- and sex-matched naive controls at the postacute time point, while young EAE mice exhibited similar brain volume compared to their appropriate controls (Figure 4H). We observed similar differences when normalizing to body weight or length of the entire skull (data not shown). Moreover, when expressed as a percentage change from the mean brain volume of age-matched controls, old but not young SJL/J A/T EAE mice at the postacute stage exhibited a significant decrease from controls (Figure 4I). Last, a strong negative correlation was observed between brain volume and EAE severity (Figure 4J). We also noted a significant decrease in somatosensory cortex volume between old SJL/J A/T EAE mice and their age-matched controls at the postacute time point (Figure 4K). These data suggest that severe, prolonged EAE results in diminished brain volume in old SJL/J A/T EAE mice, detectable by MRI as early as day 40 post-A/T.

### Aging does not affect CNS-resident T cells in SJL/J A/T EAE.

The divergent clinical and pathological (gray matter) phenotype in the old versus young SJL/J A/T EAE model emerges after the peak of disease. Thus, examining the composition of immune cells in the leptomeninges at peak disease provides an opportunity to assess what may be responsible for the subsequent poor outcome in old recipient mice. We first asked whether old versus young mice differed in the composition and phenotype of T cells at peak disease by performing flow cytometry on whole brains and spinal cords. We found no differences in the frequencies and absolute numbers of CD4^+^CD8^+^ T cells derived from the brain and spinal cord of old versus young SJL/J A/T EAE mice (Supplemental Figure 3, A–D). Furthermore, ex vivo restimulation of T cells from whole brains and spinal cords of old versus young SJL/J A/T EAE mice taken at the acute time point revealed no difference in their capacity to produce IL-17, GM-CSF, and IFN-γ (Supplemental Figure 3, E and F, and Supplemental Figure 4). These data show that differences in CNS-resident T cells are not likely to account for altered clinical and pathological attributes of SJL/J A/T EAE in old versus young mice.

### Old SJL/J A/T EAE mice exhibit a deficit of B cells and monocytes and an accumulation of neutrophils in the leptomeninges.

**Figure 5:**
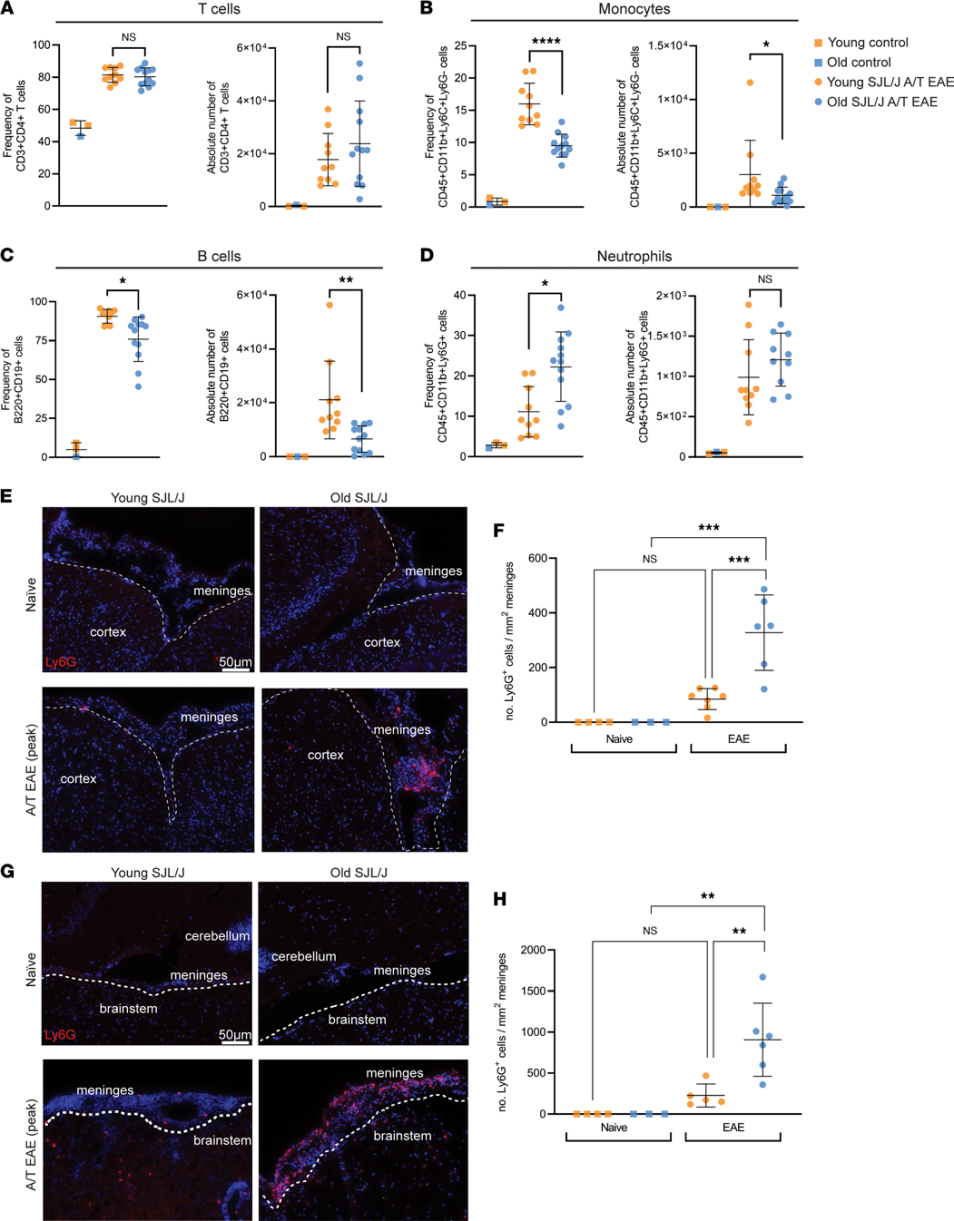
Flow cytometry and immunofluorescence of the brain from old versus young SJL/J A/T EAE mice reveal differences in immune cell compositions. Leptomeninges were separately dissected from mice at the acute time point of EAE and subjected to analysis by flow cytometry. Despite no change in (**A**) T cell number or frequency, we observed a decrease in density of (**B**) monocytes and (**C**) B cells and an increase in frequency of (**D**) neutrophils. Naive samples represent a pool of 2 young, 1 old age-matched, unimmunized controls. (**A**–**D**) Statistical analysis by 1-way ANOVA (absolute number) or Kruskal-Wallis (frequency) with correction for multiple comparisons. (**F** and **H**) Statistical analysis by Kruskal-Wallis with correction for multiple comparisons. Data are expressed as mean ± SD. We also performed immunofluorescence staining for Ly6G, identifying neutrophils in fresh-frozen brain tissue from old versus young naive and SJL/J A/T EAE mice. Note the enrichment in Ly6G^+^ neutrophils in the leptomeninges overlying the (**E** and **F**) cortex and (**G** and **H**) brainstem. *P ≤ 0.05, **P ≤ 0.01, ***P ≤ 0.001, ****P ≤ 0.0001. Scale bars: 50 μm.

Using the whole brain to ascertain the impact of age on T cell phenotype in the context of SJL/J A/T EAE may have obscured brain compartment–specific effects. Indeed, we know that T cells accumulate in the leptomeninges in both old and young SJL/J A/T EAE mice, and the leptomeninges represent only a fraction of the entire brain. To interrogate differences in compartment-specific populations, we applied flow cytometry to single-cell suspensions released from separately dissected leptomeninges and cortex and brainstem fractions, focusing again on the peak acute disease time point prior to the bifurcation of clinical phenotypes in old versus young mice in order to gain insight into what may drive nonremitting EAE. Despite separating by brain region, we still found no differences in CD3^+^CD4^+^ T cell numbers or frequencies in the leptomeninges, cortex, or brainstem, confirming our earlier results on the whole brain (Figure 5A and Supplemental Figure 5, A and E). However, in the leptomeninges we observed a 1.5-fold decrease in CD11b^+^Ly6C^+^ monocytes (*P* < 0.0001) (Figure 5B) and a 3-fold decrease in absolute number (*P* = 0.0072) and frequency (*P* = 0.0314) of CD19^+^B220^+^ B cells in the leptomeninges of old SJL/J A/T EAE mice compared with young mice (Figure 5C). We also observed a 2-fold increase in frequency of CD11b^+^Ly6G^+^ neutrophils (*P* < 0.005) (Figure 5D). When examining the brain parenchyma (cortex and brainstem), we only noted a significant decrease in B cells, a slight trend toward increase in frequency and absolute number of neutrophils, and no difference in frequencies or absolute number of monocytes (Supplemental Figure 4 and Supplemental Figure 5, B–H). Thus, alterations in monocytes, B cells, and neutrophils in old versus young recipient mice during the acute disease time point are largely restricted to the leptomeninges. To further confirm an accumulation of neutrophils in the brains of old SJL/J A/T EAE mice, we performed IF, staining for Ly6G, a marker of neutrophils. Indeed, we observed an accumulation of Ly6G^+^ cells in the leptomeninges overlying the cortices (mean old = 327.6 cells, mean young = 84.8 Ly6G^+^ cells/mm^2^ of leptomeninges, *P* < 0.001) and brainstems (mean old = 907.2 cells, mean young = 226.7 Ly6G^+^ cells/mm^2^ of leptomeninges, *P* < 0.01) of old mice (Figure 5, E–H).

Collectively, these data demonstrate that although aging does not affect the number or phenotype of brain-resident CD3^+^CD4^+^ T cells, there is a significant age-dependent difference in the accumulation of leptomeningeal B cells, neutrophils, and monocytes in the context of SJL/J A/T EAE.

### Transcriptomic analysis of the EAE leptomeninges reveals gene expression differences between old and young SJL/J A/T EAE mice.

**Figure 6:**
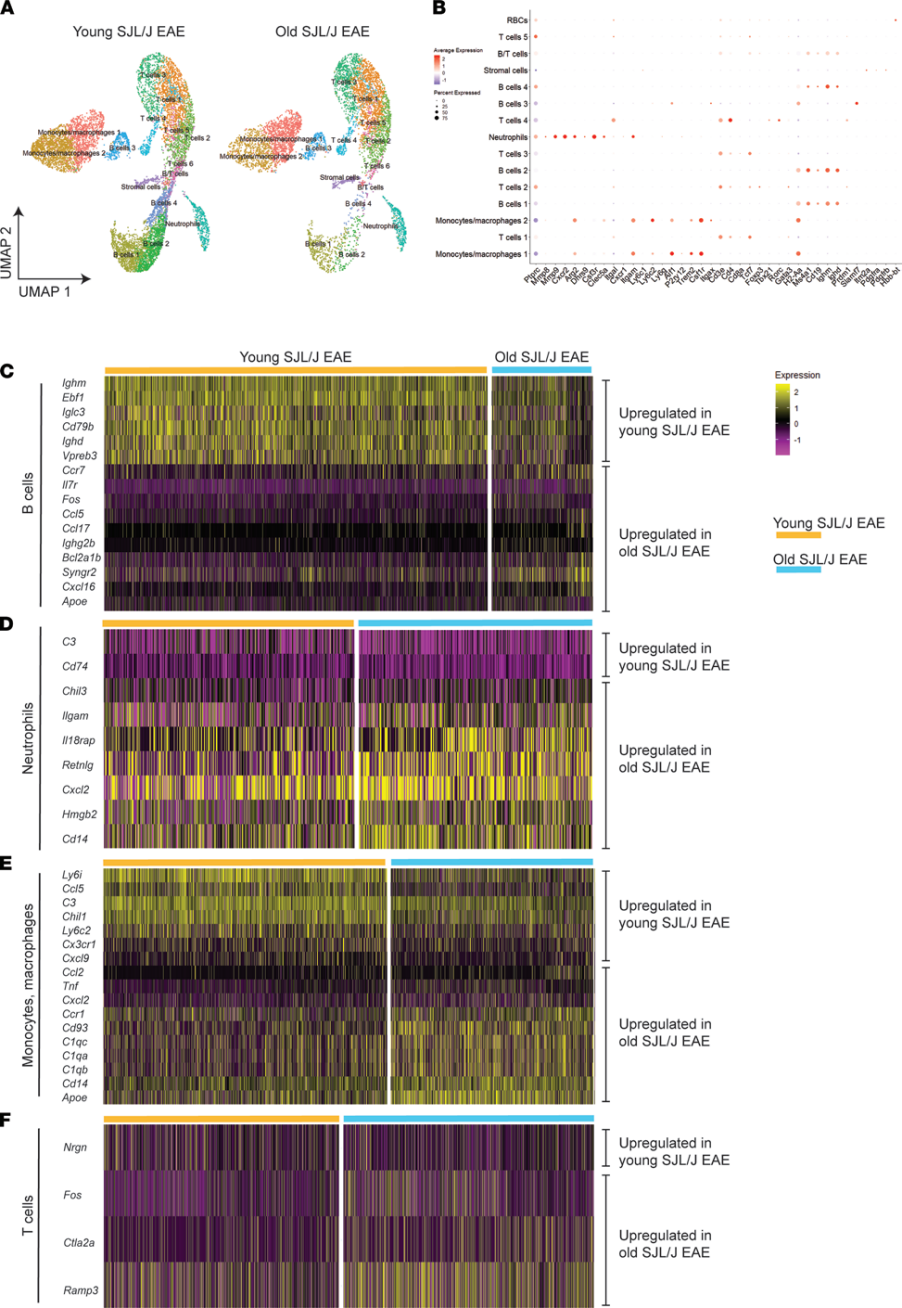
Transcriptomic analysis of the SJL/J A/T EAE leptomeninges reveals age-dependent heterogeneity in transcriptional profiles of B cell, neutrophil, and monocyte/macrophage populations at peak disease stage. Leptomeninges of old (*n* = 2) versus young (*n* = 2) SJL/J A/T EAE mice at peak disease were dissected and sent for single-cell RNA sequencing on the 10x Genomics platform. Data shown represent 2 biological and experimental repeats, each with an *n* of 1 for each group analyzed. (**A**) UMAP clustering reveals proportional differences in leptomeningeal immune cell populations. (**B**) Identification of clusters was performed using transcripts for lineage-specific markers. Differential gene expression analysis based on age was performed on (**C**) B cell, (**D**) neutrophil, and (**E**) monocyte/macrophage clusters and (**F**) T cells. Gene expression heatmaps were generated from select transcripts that exhibited a *P* < 0.01 and log_2_ fold-change cutoff of 0.5.

We next asked whether the transcriptomic landscapes of immune cells differed in the leptomeninges from old versus young mice at peak disease using single-cell RNA sequencing (scRNA-Seq). We accomplished this by submitting single-cell suspensions derived from the leptomeninges of old versus young SJL/J A/T EAE mice to sequencing on the 10x Genomics platform. We obtained a total of 18,321 cells from 2 independent experimental repeats (data available: Gene Expression Omnibus accession number GSE201568). After performing quality control and data integration pipelines in Seurat V3.0 (29), we subjected 15,641 cells to unsupervised uniform manifold approximation and projection (UMAP) clustering and identified 15 clusters based on differential gene expression (Figure 6A). We ascribed putative identities of these clusters based on dominant cluster-specific genes, including those for neutrophils (*Mmp8*, *Mmp9*, *Cxcr2*, *Ly6g*), macrophages/monocytes (*Itgam*, *Ly6c1*, *Ly6c*), T cells (*Cd3e*, *Cd4*, *Cd8a*, *Tcf7*), and B cells (*Cd19*, *Ighm*, *Ighd*) (Figure 6B). We then interrogated the gene expression profiles of B cell, neutrophil, and monocyte clusters because these populations were differentially represented in old versus young mice by flow cytometry. We noted 495 differentially expressed genes among the B cell clusters, 473 genes in the neutrophil cluster, 107 genes among the monocyte/macrophage clusters, and only 82 genes among the T cell clusters in old versus young SJL/J A/T EAE mice (Supplemental Data 1). We then filtered for genes with a *P* value of less than 0.01. In the B cell clusters, we found that young EAE mice upregulated more transcripts involved in B cell development, such as *Ighm*, *Ebf1*, *Iglc3*, *Cd79b*, *Ighd*, and *Vpreb3*, while old EAE mice upregulated proinflammatory genes, such as *Il7r*, *Fos*, *Ccl5*, *Ccl17*, *Ighg2b*, *Bcl2a1b*, *Syngr2*, *Cxcl16*, and *Apoe* (Figure 6C). Of the genes in the neutrophil clusters, we found that young EAE mice expressed more transcripts for *C3* and *Cd74*, while old EAE mice expressed more transcripts associated with innate immunity, such as *Chil3*, *Itgam*, *Il18rap*, *Retnlg*, *Cxcl2*, *Hmgb2*, and *Cd14* (Figure 6D). Last, in the monocyte/macrophage clusters, we found young EAE mice had higher levels of transcripts for *Ly6i*, *Ccl5*, *C3*, *Chil1*, *Ly6c2*, *Cx3cr1*, and *Cxcl9*, while old EAE mice had higher levels of transcripts for inflammation and complement pathways, such as *Ccl2*, *Tnf*, *Ccr1*, *Cd93*, *C1qc*, *C1qa*, *C1qb*, *Cd14*, and *Apoe* (Figure 6E). Of the T cells, young EAE mice expressed higher levels of *Nrgn*, while old EAE mice expressed high levels of *Fos*, *Ctla2a*, and *Ramp3* (Figure 6F). These data further suggest that while T cells are important in establishing disease and initiating pathogenesis, other cells such as B cells, neutrophils, and monocytes/macrophages contribute to the differential clinical courses between old and young SJL/J A/T EAE mice.

### Neutrophils populate the leptomeninges of PMS brains.

**Figure 7:**
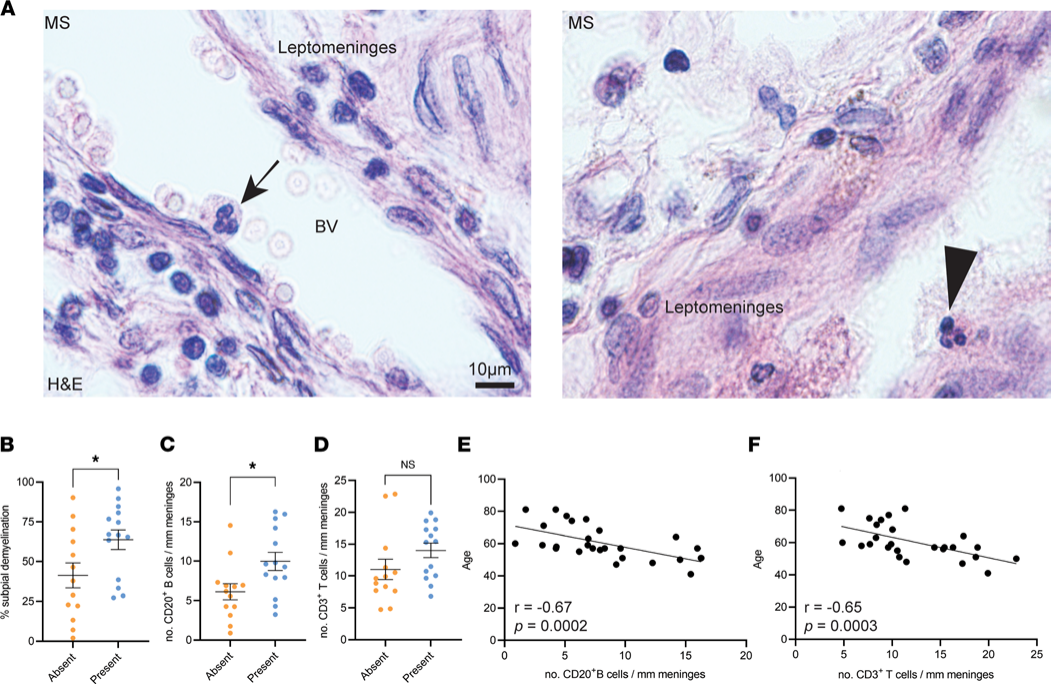
The presence of neutrophils in the leptomeninges of patients with PMS is associated with more extensive subpial demyelination and a higher density of leptomeningeal B cells. (**A**) Representative hematoxylin and eosin (H&E) staining of formalin-fixed paraffin-embedded cortex from PMS patients (*n* = 27) showing a neutrophil localized within a blood vessel (BV) (arrow) and a neutrophil localized outside of blood vessels (arrowhead) in the leptomeninges lining the cortex. Quantification of (**B**) percentage subpial demyelination, (**C**) density of meningeal CD20^+^ B cells, and (**D**) density of meningeal CD3^+^ T cells in MS donors with or without neutrophils identified outside BVs in the leptomeninges. Statistical analysis by unpaired Student’s 2-tailed *t* test; error bars indicate mean ± SEM. (**E**) Correlation analyses between age of donor and number of CD20^+^ cells reveals negative correlation (*r* = –0.67, *P* = 0.0002) between age and B cells. statistical analysis by Spearman’s correlation test. (**F**) Correlation analyses between age of donor and number of CD3^+^ cells reveals negative correlation (*r* = –0.65, *P* = 0.0003) between age and T cells. Statistical analysis by Spearman’s correlation test. *P ≤ 0.05, **P ≤ 0.01.

Neutrophils have been implicated in demyelination and axonal degeneration during the acute phase of EAE in C57BL/6 mice (30), but little is known about their presence in the MS brain. We therefore examined previously characterized postmortem MS brains, which showed a range of subpial demyelination and meningeal inflammation compared with age-matched non-neurological controls (31), for the presence of neutrophils in the leptomeningeal compartment using high-magnification microscopy on H&E-stained samples. Neutrophils were identified based on their multilobular nuclei. Although they were rare, we found neutrophils in the leptomeninges of a subset of MS donors (Figure 7A). We then stratified donors based on the presence or absence of neutrophils in the leptomeninges and performed correlation studies comparing the extent of subpial demyelination and leptomeningeal inflammation with the presence or absence of neutrophils. The donors with leptomeningeal neutrophils exhibited a higher percentage of demyelination in the subpial cortex compared with donors without leptomeningeal neutrophils (63.8% versus 41.3% demyelination, *P* < 0.05) (Figure 7B), as well as a higher number of leptomeningeal CD20^+^ B cells (10.0 CD20^+^ cells/mm length of leptomeninges versus 6.1 CD20^+^ cells/mm length of leptomeninges) (Figure 7C). We did not observe an association of leptomeningeal T cells with the presence or absence of neutrophils (Figure 7D). These changes were not linked to a particular MS subtype since the density of CD20^+^ B cells or the density of CD3^+^ T cells did not differ significantly between patients with primary progressive MS versus secondary progressive MS (SPMS) (average number of CD20^+^ cells/mm length of leptomeninges: 6.7 vs. 9.1; average number of CD3^+^ cells/mm length of leptomeninges: 12.2 vs. 13.4). In line with a previous report where inflammation in the MS brain was reduced with increasing age (32), we also noted a decrease in leptomeningeal inflammation with increasing age (CD20^+^ B cells/mm meninges: Spearman’s *r* = –0.67, *P* = 0.0002; CD3^+^ T cells/mm meninges: Spearman’s *r* = –0.65, *P* = 0.0003) (Figure 7, E and F). In conclusion, we identified neutrophil accumulation in the leptomeninges of a subset of patients with PMS who also exhibited leptomeningeal inflammation and cortical subpial demyelination.

## Discussion

In this study, we show that A/T of PLP-primed encephalitogenic Th17 cells into old SJL/J mice induces nonremitting EAE. Furthermore, the nonremitting clinical course was accompanied by leptomeningeal inflammation, gray matter demyelination, axonal damage, synapse loss, and disruption of the glial limitans, as well as brain atrophy and accumulation of NfL in the serum, all hallmarks of PMS. In the absence of EAE, as we did not observe a decrease in brain volume with age, which is in contrast to the findings of Taylor et al. (33). However, Taylor et al. examined brain volume in 24-month-old C57BL/6 mice, whereas our old SJL/J mice were 8 months old. These differences in age and genetic background of mice may account for this discrepancy.

We also show that this model is highly reproducible across vivaria and is independent of sex of recipient mice, providing further evidence that age is the primary driver of the clinical and pathological phenotype. Therefore, A/T of encephalitogenic Th17 cells into old versus young SJL/J mice is a valuable method for ascertaining the role of aging on gray matter pathology associated with leptomeningeal inflammation in EAE in the absence of the confounding adjuvant-driven/cytokine storm effects that occur with active EAE or lentivirally based introduction of cytokines (30, 34, 35).

We have previously shown that cytokines such as IL-17A, IL-17F, IL-22, and lymphotoxin exert direct effects on the underlying stromal cells of the leptomeningeal subarachnoid space, resulting in the release of chemokines and cytokines that in turn recruit and tune additional immune cells (T cells, B cells, myeloid cells, and so on) that infiltrate the leptomeninges. This results in the formation of so-called TLTs that exhibit varying degrees of complexity and organization (23). Although these structures are also present in early MS (9), they may play a particularly pathogenic role in the progressive phase of the disease (20, 36). Thus, one potential reason for inefficacy of immunomodulatory therapies in PMS (37) could be due to their lack of access to these structures and/or redundant immune-mediated mechanisms that sustain these structures. Alternatively, it is possible that leptomeningeal TLT cannot be silenced by a single therapy. Since these structures are evident from the earliest stages of MS, we reason that they may be fueling a form of “silent progression” that ultimately impacts the health of the underlying gray matter.

While age is the strongest predictor of MS progression (25), we do not know what aspects of aging affect the MS brain such that it becomes more susceptible to gray matter injury. One possible explanation for the severe EAE phenotype in old SJL/J recipient mice is that young Th17 cells become more activated in the environment of the old CNS. However, we found no difference in the quantity or quality of CNS-resident CD4^+^ T cells, suggesting that other age-associated changes are dominant factors that determine clinical outcome. Instead, we noted a paucity of monocytes/B cells and an increase in neutrophils in the leptomeninges overlying the cortex and brainstem of old SJL/J A/T EAE mice compared with young SJL/J A/T EAE mice by flow cytometry and IF microscopy. These experiments were performed at the peak of disease, prior to the bifurcation of the old versus young clinical phenotype. Thus, changes at the acute phase may “set up” the CNS for postacute clinical disease. We did note that while there was no significant difference in absolute number of meningeal neutrophils between old and young mice measured by flow cytometry, the density of neutrophils measured by IF in old mice was higher in the leptomeninges overlying cortex (*P* < 0.001) and in the leptomeninges overlying the brainstem (*P* < 0.01) compared with young mice. This may be due to the fact that the entire meninges are stripped from the brain for flow cytometry, whereas for IF, the quantification is focused on foci of immune cell aggregates. Therefore, it is possible that the enrichment in neutrophils detected in old mice by IF is diluted when measured across the entirety of the leptomeninges by flow.

To gain insight into the functional states of each cellular compartment associated with the remitting or nonremitting disease course in SJL/J A/T EAE mice, we chose a single-cell transcriptomic approach. We interrogated differences in gene expression of leptomeningeal T cells, B cells, neutrophils, and monocytes/macrophages of old versus young SJL/J A/T EAE mice at peak disease. While T cell clusters showed a similar transcriptional signature, the B cell lineage showed an upregulation of several genes associated with developing B cells in young mice (*Ebf1*, *Vpreb3*, *Cd79b*, *Ighm*, *Ighd*), while more genes associated with mature and inflammatory B cells were upregulated in old mice (*Ighg2b*, *Bcl2a1b*, *Fos*, *Cxcl16*, *Ccl5*, *Ccr7*). Within the neutrophil cluster, we found an upregulation of genes associated with phagocytosis and immune activation (*Cd14*, *Itgam*, *Hmgb2*) as well as an upregulation of transcripts for the neutrophil chemoattractant *Cxcl2*, which has been reported in the literature to be expressed by activated neutrophils to facilitate recruitment of more neutrophils (38, 39). In the monocyte/macrophage clusters, we found cells from old mice exhibited an upregulation of transcripts for inflammatory cytokines (*Cxcl2*, *Tnf*, *Ccl2*) and phagocytosis (*Cd14*, *Apoe*) as well as the classical complement pathway (*C1qa*, *C1qb*, *C1qc*, *Cd93*). The latter is known to be involved in the stripping of synapses (40, 41) and could therefore be a potential mechanism at play in the brain of old EAE mice where we observed a loss of synapses in the cortex.

An important question is whether our model is generalizable across genetic backgrounds and more broadly relevant to MS disease progression. Although C57BL/6 mice exhibit predominantly spinal cord pathology, Segal and colleagues have recently shown that age is associated in that strain with a nonremitting phenotype that bears many similarities to what we observe here in SJL/J mice (42), suggesting that age is a powerful modifier of CNS pathology independent of strain differences. In comparing this model to human MS, not only are many features of progressive MS recapitulated, but also aged A/T EAE SJL/J EAE mice demonstrated leptomeningeal accumulation of neutrophils, a finding we validated in the leptomeninges of a subset of patients with SPMS who also exhibited leptomeningeal inflammation and cortical demyelination. Neutrophils have likely been underestimated in MS studies, as they are innate immune cells, typically short-lived, and relatively scarce in MS postmortem brain tissues with long-standing disease (43). However, neutrophils may contribute to MS and EAE pathogenesis in several ways. Yamasaki et al. have shown that in the C57BL/6 model of EAE, neutrophils are capable of engulfing myelin and contributing to demyelination (44). Neutrophils also secrete a repertoire of inflammatory mediators, including IL-1β (45, 46), which may stimulate differentiation of CD4^+^ T cells into Th17 cells. Neutrophils also produce matrix metalloproteinases and myeloperoxidases, which may contribute to blood-brain barrier leakage and breakdown(47, 48). Indeed, in our scRNA-Seq data, we detected transcripts for *Mmp8* and *Mmp9* as well as *Il1b* in the leptomeningeal neutrophil cell cluster, suggesting that neutrophils in our SJL/J A/T EAE model may be involved in mediating stromal remodeling and inflammation. It is important to note that while our EAE studies showed an enrichment of neutrophils and a paucity of CD20^+^ B cells in the brain leptomeninges at peak disease, the MS cohort analyzed showed that donors with neutrophils in the meninges had an enrichment of CD20^+^ lymphocytes in the leptomeninges. This apparent discrepancy may be due to the fact that the human tissue analyzed in this study is from a cohort of PMS patients with long-standing disease (>20 years) as opposed to the EAE model herein, which captures acute inflammatory changes at peak disease. Indeed, although present, the neutrophils in the PMS postmortem brains were rare, and B cells showed a diffuse localization throughout the leptomeninges as described in other donor cohorts (31). The effects of neutrophil depletion in our EAE model are currently under investigation and will be an important follow-up to this study.

Our study has some limitations that can be followed up in the future. Specifically, because of tissue processing biases, our scRNA-Seq and flow cytometry data are enriched for immune cells rather than stromal cell populations. We have previously shown that stromal cells in the subarachnoid space are dramatically remodeled following introduction of PLP-primed Th17 cells (23); thus, a better understanding of alterations in stromal cell gene expression in response to Th17 cell infiltration may provide clues into what drives subpial gray matter pathology. Moreover, during aging, microglia and astrocytes become more inflammatory, and neurons are increasingly prone to damage (21, 49–51). Nevertheless, the connection between inflammatory factors that originate from the leptomeninges and their impact on underlying glial cells remains relatively unexplored, and our model will serve as a valuable tool for interrogating the effects of aging on gray matter pathology.

## Methods

### Mice.

Female 6- to 10-week-old SJL/J CD45.1^+^ mice were obtained from Envigo (mouse code: 052). Animals were housed at the U of T animal facilities under specific pathogen–free conditions, in a closed caging system with a 12-hour light/12-hour dark cycle. They were provided with a standard irradiated chow diet (Teklad, Envigo, 2918) and acidified water (reverse osmosis and ultraviolet sterilized) ad libitum.

At the UBC, SJL/J mice (Envigo) were bred and housed under specific pathogen–free conditions at the Center for Disease Modeling. Up to 5 mice per cage were housed in Ehret cages with BetaChip bedding and had ad libitum access to standard irradiated chow (PicoLab Diet 5053) and reverse osmosis/chlorinated (2–3 ppm) purified water. Housing rooms were maintained on a 14-hour light/10-hour dark cycle with temperature and humidity ranges of 20°C–22°C and 40%–70%, respectively. All experiments were performed according to guidelines from UBC Animal Care Committee and Biosafety Committee–approved protocols.

### Induction of EAE and clinical evaluation.

Donor 6-week SJL/J female mice were immunized with 100 μg of PLP_139-151_ (HSLGKWLGHPDKF; Canpeptide) in an emulsion of incomplete Freund’s adjuvant (BD Difco), supplemented with 200 μg *Mycobacterium tuberculosis* H37 Ra (BD Difco, 231141) in a total volume of 300 μL administered as three 100 μL subcutaneous injections on the back and flanks. Nine days postimmunization, donors were sacrificed by CO_2_ asphyxiation. Subsequently, cells from spleens and lymph nodes (inguinal, axillary, brachial, and cervical) were released, and cells were then restimulated ex vivo with PLP_139–151_ (10 μg/mL) in the presence of anti–IFN-γ (20 μg/mL, Bioceros), anti–IL-4 (20 μg/mL, Bioceros), and IL-23 (10 ng/mL, R&D Systems) for 72 hours at 37°C. In total, 1 × 10^7^ cells were injected intraperitoneally into SJL/J recipient mice.

For clinical assessment of EAE disease, recipient mice were weighed daily and scored according to a composite scale that we and others have previously published. Briefly, the composite scale measures mobility impairments in each limb and the tail. Each limb is graded from 0 (asymptomatic) to 3 (complete paralysis), and the tail is graded from 0 (asymptomatic) to 2 (limp tail). Assessment of the righting reflex is scored from 0 to 2, with 0 being assigned for a normal righting reflex, 1 for slow righting reflex, and 2 for a delay of more than 5 seconds in the righting reflex. Each criterion was measured in 0.5 increments. Thus, the composite score ranges from 0 (nonsymptomatic) to 16 (fully quadriplegic mouse with limp tail and significantly delayed righting reflex) (52–54).

### Histology and immunostaining.

Seven-micron paraffin sagittal sections of mouse brain were collected from the midline, mounted on Superfrost Plus glass slides (Knittel Glass), and dried in the oven (Precision compact oven, Thermo Fisher Scientific) overnight at 37°C. Paraffin sections were deparaffinized in xylene (Fisher Chemical, Thermo Fisher Scientific) and rehydrated through a series of ethanol washes. Histology was performed using standard H&E stain with placement in xylene before being coverslipped with Entellan mounting media (MilliporeSigma).

For IHC, FFPE slides were deparaffinized and rehydrated as described above. Slides were subsequently incubated in 0.3% H_2_O_2_ in methanol for 20 minutes to block endogenous peroxidase activity. Epitopes were exposed by heat-induced antigen retrieval in 10 mM Tris + 1 mM EDTA (pH 9.0), depending on the antibody used (see Supplemental Table 1) in a pressure cooker placed inside a microwave set at high power (~800 W) for 20 minutes. Endogenous peroxidase activity was blocked by incubation in PBS with 0.3% H_2_O_2_ for 20 minutes at room temperature. Nonspecific protein binding was blocked by incubation with 10% normal goat serum (DAKO). The nonspecific binding of antibodies was blocked using 10% normal goat serum (DAKO) in PBS for 20 minutes at room temperature. Myelin protein was detected using an antibody for PLP, microglia/macrophages were detected using an antibody for Iba1, the glial limitans was detected using an antibody for GFAP, neurofilament was detected using an antibody for pan-neurofilament, and synapses were detected using an antibody against the neuroendocrine secretory granule membrane (synaptophysin). Primary antibodies were applied overnight at 4°C, diluted in Normal Antibody Diluent (Immunologic). The following day, sections were incubated with a post–antibody blocking solution for monoclonal antibodies (Immunologic) diluted 1:1 in PBS for 15 minutes at room temperature (RT). Detection was performed by incubating tissue sections in secondary poly-HRP goat anti-mouse/rabbit/rat IgG (Immunologic) antibodies diluted 1:1 in PBS for 30 minutes at RT followed by application of DAB (Vector Laboratories) as a chromogen. Counterstaining was performed with hematoxylin (Sigma-Aldrich) for 10 minutes. The sections were subsequently dehydrated through a series of ethyl alcohol solutions and then placed in xylene before being coverslipped with Entellan mounting media (Sigma-Aldrich). The colorimetric staining was visualized under a light microscope (Axioscope, Zeiss), connected to a digital camera (AxioCam MRc, Zeiss), and imaged with Zen pro 2.0 imaging software (Zeiss).

### Quantification of histology and IHC.

For each marker analyzed, 2–3 stained sections were scored for each mouse. To ensure that similar areas were compared across mice, sections were collected from the midline outward in each brain hemisphere. Sagittal sections were collected to ensure that many anatomical regions of the brain (from the olfactory bulbs to the brainstem) were captured at once. We screened entire brain sections under the microscope, but for the analysis we focused on areas where TLTs were usually observed (meninges outlining the somatosensory cortex, hippocampus and brainstem). Red/green/blue (RGB) images of H&E, PLP, IBA1, GFAP, pan-neurofilament, synaptophysin, and NfL stains from brain sections were acquired at original magnification ×20 using a light microscope (Axioscope, Zeiss), connected to a digital camera (AxioCam MRc, Zeiss), and Zen pro 2.0 imaging software (Zeiss). ImageJ Pro Plus 7.0 imaging software (MediaCybernetics) was used to measure the extent of demyelination, axonal loss, and synapse loss in the subpial cortex and to measure the length of uninterrupted GFAP^+^ glial limitans and the surface area analyzed in the digital images.

The quantification of subpial demyelination axonal loss and synapse loss was performed on PLP-, pan-neurofilament-, NfL-, and synaptophysin-immunostained sections, respectively, on regions of interest (ROIs) that were selected based on being adjacent or not to TLTs identified on serial sections by H&E staining. The RGB images were separated into single-color channels using the color deconvolution plugin in ImageJ. The single-color channel for PLP or pan-neurofilament or NfL or synaptophysin was subjected to thresholding to create a mask that captured the specific staining. The area fraction measurement was applied to each ROI to quantify the percentage of thresholded staining. The scale represents the percentage of PLP^+^ or pan-neurofilament^+^ or NfL^+^ or synaptophysin^+^ subpial area in each brain section examined.

The density of IBA1^+^ cells was determined using a morphometric grid (at original magnification ×200), and the data were expressed as cells per mm^2^.

The extent of uninterrupted glial limitans was determined using the measurement plugin in ImageJ, and data were expressed as percentage of total glial limitans scored in the images.

For the histological assessment of TLTs, a quantification of TLTs’ numbers per mouse and size (TLT area/TLT) was performed on the H&E-stained brain sections. In addition, the extent of B220^+^ B cells and CD3^+^ T cells’ accumulation in the TLTs were scored using a semiquantitative scale with 0 = no cells, 1 = thin layer of cells, 2 = multiple layers of cells. For all quantifications of staining, the experimental groups were blinded to the investigators.

### T cell stimulation.

Whole brains and spinal cords were mashed in digestion buffer (10 mM HEPES, 150 mM NaCl, 1 mM MgCl_2_, 5 mM KCl, and 1.8 mM CaCl_2_ in HBSS buffer). To dissociate cells from their resident tissues, collagenase D (Roche) was added to a final concentration of 1 mg/mL and DNAse I (Roche) to a final concentration of 60 μg/mL to each sample. CNS samples were incubated at 37°C for 30 minutes, mixed with a pipette tip, and reincubated for an additional 15 minutes. Upon removal, a final concentration of 1 mM EDTA pH 8.0 was added to each sample and incubated at RT for 10 minutes. Samples were then filtered through a 70 μm filter and washed twice with ice-cold PBS. Cells were resuspended into a 30% Percoll (GE Healthcare) solution and centrifuged to separate the fat from the cells. Collected lymphocytes were washed twice in ice-cold PBS and resuspended in complete RPMI (10% FBS from Gibco, Thermo Fisher Scientific; l-glutamine from MilliporeSigma; sodium pyruvate from MilliporeSigma; penicillin from MilliporeSigma; streptomycin from MilliporeSigma; HEPES pH 7.0 from Gibco, Thermo Fisher Scientific; and β-mercaptoethanol from Gibco, Thermo Fisher Scientific; in RPMI-1640 medium from MilliporeSigma). Whole brains and spinal cords were dissected and digested as described earlier. Cells were counted with a hemocytometer and plated at a density of 250,000 cells/well. Following incubation at 37°C for 5 hours with an intracellular cytokine restimulation buffer (PMA [MilliporeSigma, stock 500 μg/mL] used at 1:100,000; ionomycin [MilliporeSigma, stock 0.5 mg/mL] used at 1:1000; and Brefeldin A [eBioscience, Thermo Fisher Scientific, stock 100x] used at 1:1000 in complete RPMI), cells were collected, washed twice in ice-cold PBS, and stained for flow cytometry.

### Flow cytometry.

Single-cell suspensions from CNS tissues were stained for viability with aqua, washed, and subsequently surface-stained with a panel of fluorescently conjugated antibodies against CD45.1 (A20), CD3 (17A2), CD4 (RM4-5), CD19 (1D3), B220 (RA3-6B2), Gr-1 (RB6-8C5) or Ly6G (1A8), Ly6C (HK1.4), CD11b (M1/70), and CD11c (N418). Cell suspensions from T cell stimulation were surfaced-stained for CD4 (RM4-5), permeabilized with CytoFix/CytoPerm (BD Biosciences) for 20 minutes at 4°C, and subsequently stained intracellularly with GM-CSF (eBioscience, Thermo Fisher Scientific), IFN-γ (eBioscience, Thermo Fisher Scientific), and IL-17A (eBioscience, Thermo Fisher Scientific) (Supplemental Table 1). Cells were acquired on a BD LSR X20 using FACSDiva software.

### Single-cell isolation from spinal cord dissections.

Mice were sacrificed by CO_2_ asphyxiation were decapitated, and skin overlying the vertebral column was removed. Spinal cords were removed by cutting open the vertebral column and transferred to 6-well plates containing 3 mL of digestion buffer (10 mM HEPES, 150 mM NaCl, 1 mM MgCl_2_, 5 mM KCl, and 1.8 mM CaCl_2_ in HBSS buffer) containing DNAse I (60 μg/mL; Roche) and collagenase D (1 mg/mL; Roche). Digestion was allowed to proceed at 37°C, 5% CO_2_ for 30 minutes. The cells were subjected to 30% Percoll (GE Healthcare) gradient purification before downstream applications such as flow cytometry and T cell stimulation.

### Single-cell isolation from leptomeningeal and cortical dissections.

Mice were sacrificed by CO_2_ asphyxiation and were decapitated, and skin overlying the skull was removed. Skull caps were carefully separated from the brain to remove dura mater, and brains were transferred to Petri dishes containing 1 mL of ice-cold PBS. Under a dissection microscope, leptomeninges were removed from the brainstem, cerebellum, ventricles, hypothalamus, and cortex into digestion buffer (10 mM HEPES, 150 mM NaCl, 1 mM MgCl_2_, 5 mM KCl, and 1.8 mM CaCl_2_ in HBSS buffer) containing DNAse I (60 μg/mL) and collagenase D (1 mg/mL). Cortices were removed by first bisecting the brain along the sagittal plane to expose the corpus callosum, followed by removal of the brainstem, midbrain, cerebellum, and hypothalamus. Finally, cortices were isolated by dissecting away the corpus callosum and thalamus, and placed into digestion buffer. Cortices were mechanically dissociated by finely chopping via scalpel blade and then subjected to digestion with DNAse I and collagenase D for 30 minutes at 37°C, 5% CO_2_. Cells from both leptomeninges and cortical digestions were subjected to 30% Percoll (GE Healthcare) gradient purification before downstream applications such as scRNA-Seq.

### scRNA-Seq.

Prior to euthanasia, old and young EAE and naive SJL/J mice were injected intravenously with 3 μg of anti-CD45-PE (eBioscience, Thermo Fisher Scientific) to label blood-derived and blood vessel–adjacent immune cells. Leptomeninges and cortices were dissected and single-cell suspensions were prepared as previously described. Cells from each individual mouse were stained using BioLegend TotalSeq B hashtags (Hashtags 1–4, catalog numbers 155831, 155833, 155835, 155837) and an anti-PE oligonucleotide barcode (BioLegend, catalog 408113). Cells from each compartment (i.e., leptomeninges) were mixed in a 1:1 ratio, resuspended to a concentration of 1300 cells/μL, and submitted for sequencing on the 10x Genomics platform using 5′ chemistry at the Princess Margaret Genomics Centre in Toronto, Ontario, Canada. Data are available in the NCBI Gene Expression Omnibus database (accession number: GSE201568).

### Single molecular array assay for neurofilament light chain.

The amount of NfL in mouse serum was quantified with a single-molecule array (Simoa) NF-light assay (Quanterix). In brief, magnetic beads were conjugated with monoclonal capture antibodies (mAB47:3, UmanDiagnostics), then incubated with diluted mouse serum (1:8 or 1:16 dilution) and biotinylated detection antibodies (mAB2:1, UmanDiagnostics). Upon adding streptavidin-conjugated β-galactosidase (Quanterix), Resorufin β-D-galactopyranoside (Quanterix) was added for detection. The experiment was performed on a Simoa HD-X Analyzer (Quanterix). The assay was performed in duplicates, and the mean of the 2 measured serum NfL values per sample is reported.

### Preparation of samples for MRI.

Mice destined for MRI were sacrificed by CO_2_ asphyxiation and transcardially perfused using a peristaltic pump at a rate of 1 mL/min. Mice were first perfused with 40 mL of PBS containing 2 mM ProHance (Bracco Diagnostics) and 400 USP heparin (Fresnius Kabi), followed by 30 mL of PBS containing 2 mM ProHance and 4% paraformaldehyde (EMS). Skulls were decapitated and placed into PBS containing 2 mM ProHance and 4% paraformaldehyde (EMS). After an overnight incubation at 4°C, skulls were transferred to PBS containing 2 mM ProHance with 0.02% sodium azide (Fisher Scientific). Following 30-day incubation, skulls were scanned for MRI at the Mouse Imaging Centre in The Centre for Phenogenomics in Toronto, Ontario, Canada.

### Anatomical image acquisition.

A 7-Tesla 306 mm horizontal-bore magnet (BioSpec 70/30 USR, Bruker) with a ParaVision 6.0.1 console (55) was used to image brains in skulls. Eight samples were imaged in parallel using a custom-built 8-coil solenoid array. Anatomical image acquisition was modeled from Spencer Noakes et al. (56) with the following scan parameters: T2W 3D FSE cylindrical *k*-space acquisition sequence, TR/TE/ETL = 350 ms/12 ms/6, TEeff = 30 ms, 4 effective averages, FOV/matrix size = 20.2 × 20.2 × 25.2 mm/504 × 504 × 630, total imaging time = 13.2 hours. The resulting anatomical images had an isotropic resolution of 40 μm voxels.

### MRI registration and analysis.

To assess any changes to the mouse brains due to age and treatment, all anatomical brain images were registered together using the mni_autoreg (57) and Advanced Normalizations Tools (58) tool kits. The resulting consensus average and Jacobian determinants were used to quantifying volumetric differences between each MRI image and the average. The MAGeT pipeline (59) was used to segment images using a published classified MRI.

### Postmortem tissue retrieval.

Tissue blocks for this study were obtained from the Netherlands Brain Bank (Amsterdam, the Netherlands). For the characterization of leptomeningeal immune cells, tissue blocks from 27 donors with progressive (primary progressive or secondary progressive) MS were selected based on the presence of leptomeninges adjacent to the cortex in the tissue blocks. Tissue blocks were dissected based on the identification of lesions as guided by macroscopical examination and/or by postmortem MRI (since 2001) of 1 cm thick coronal brain slices (60). The tissue blocks used for the analysis of leptomeningeal inflammation and subpial demyelination performed in this study were dissected from the supratentorial cortex at locations that included the occipital or the parietal or the temporal or the frontal lobes.

Detailed clinical-pathological and demographic data of all donors are provided in Supplemental Table 2. The age at the time of death of MS patients ranged from 41 to 81 years (median: 58 years), with a mean postmortem delay of 8 hours and 31 minutes (SD, ± 1 hour 42 minutes). The clinical diagnosis of MS and its clinical course were determined by a certified neurologist and confirmed by a certified neuropathologist based on the neuropathological analysis of the patient’s brain autopsy.

### Neuropathological techniques and IHC.

For the classification of cortical gray matter lesions, sections were stained by IHC for the PLP marker of myelin. For the identification of neutrophils, sections were stained with H&E. Leptomeningeal immune cells were identified by IHC for CD3 to detect T cells and CD20 to detect B cells (Supplemental Table 1).

IHC was performed as previously described (32, 61). Sections of 7 μm thickness were cut from FFPE tissue blocks, collected on Superfrost Plus glass slides (VWR international), and dried overnight at 37°C. Sections were deparaffinized in xylene (2 × 15 minutes) and rehydrated through a series (100%, 70%, 50%) of ethanol. Endogenous peroxidase activity was blocked by incubation in methanol (Merck KGaA) with 0.3% H_2_O_2_ (Merck KGaA) for 20 minutes at RT. Sections were then rinsed in PBS and pretreated with microwave antigen retrieval (3 minutes at 900 W followed by 10 minutes at 90 W) in either 0.05 M Tris-buffered saline (pH 7.6) or 10 mM Tris/1 mM EDTA buffer pH 9.0 (Supplemental Table 1).

Sections were incubated overnight at 4°C in the appropriate primary antibody (Supplemental Table 1) diluted in Normal Antibody Diluent (Immunologic) and the next day with the BrightVision poly-HRP-Anti Ms/Rb/Rt IgG biotin-free (diluted 1:1 in PBS, Immunologic) for 30 minutes at RT. The immunostaining was visualized with DAB (Vector Laboratories) for 4 minutes at RT, and sections were counterstained with hematoxylin (Sigma Chemie GmbH), dehydrated in ethanol, and mounted with Pertex (Histolab).

### Quantification of subpial demyelination and leptomeningeal inflammation.

For the quantification of subpial demyelination, brain slides stained for PLP identifying myelin were used. Subpial (type III) lesions — which span from the pial surface typically to cortical layers 2–4 — were imaged at original magnification ×5 using a light microscope (Axioscope), connected to a digital camera (AxioCam MRc), and Zen pro 2.0 imaging software. The RGB images were separated into single-color channels using the color deconvolution plugin in ImageJ Pro Plus 7.0 imaging software (MediaCybernetics). The single-color channel for PLP was subjected to thresholding to create a mask that captured the specific staining. The area fraction measurement was applied to each image to quantify the percentage of thresholded staining. The scale represents the percentage of PLP^+^ subpial cortical area in each brain section examined. For the quantification of leptomeningeal inflammation, leptomeningeal segments were randomly selected for imaging at 20× original magnification with a light microscope (Olympus BX41TF) connected to the Cell D software (Olympus). Immune cells were quantified in leptomeningeal areas that were adjacent to type III (subpial) gray matter lesions (GMLs) and in areas that were adjacent to normal appearing gray matter (NAGM). A total of 71% ± 20% (mean ± SD) of intact leptomeninges were available for scoring in the MS cohort. CD20^+^ B cell counts were done over a total leptomeningeal area of 47.53 mm^2^ from MS patients, of which 37.02 mm^2^ was adjacent to GMLs and 10.51 mm^2^ was adjacent to NAGM; and 5.652 mm^2^ of leptomeningeal area was adjacent to non-neurological control cortex. CD3^+^ T cell counts were done in a total leptomeningeal area of 60.97 mm^2^ from patients, of which 48.3 mm^2^ was adjacent to GMLs and 12.67mm^2^ was adjacent to NAGM. The leptomeningeal area (in mm^2^) was measured using the “measurement” function of the Image Pro Plus 7.0 imaging software. Cell numbers were expressed as mean number per mm of intact leptomeninges.

### Statistics.

Unless otherwise stated, all statistical tests were run using GraphPad Prism v8.0. All quantification data were subjected to Shapiro-Wilk normality test. Only *P* values less than 0.05 were considered significant. Flow cytometry data were analyzed using FlowJo v17.0. scRNA-Seq data were processed using the CellRanger (v6.1.1) feature barcoding pipeline, and read matrices were analyzed in R (v3.0) using the Seurat package (v3.0) (29). The statistical tests used for each experimental data set are indicated in the text and figure legends.

### Study approval.

All postmortem human tissue was collected with informed consent for the use of material and clinical data for research purposes, in compliance with ethical guidelines of the Vrij Universiteit and Netherlands Brain Bank, Amsterdam, the Netherlands (Reference 2009/148). In addition, the U of T Research Ethics Board granted approval for conducting histology on all postmortem human tissue (study number: 36850). All animal experiments were conducted in accordance with institutional guidelines, with ethical approval from the U of T Faculty of Medicine Animal Care Committee.

## Author contributions

MZ, NMF, XIM, AP, DSWL, LAW, and AS carried out the A/T SJL/J EAE induction protocol for various experiments. MZ, VR, and LAW maintained the young and old SJL/J mouse colonies. MZ and LAW performed the flow cytometry experiments. MZ and AP performed the scRNA-Seq experiments. MZ and SS carried out the MRI experiments. MZ analyzed the flow cytometry, scRNA-Seq, and MRI data, together with JLG. JK and EP carried out the serum NfL assay experiments and participated in the data analysis. VR and MZ performed the immunostaining experiments and data analysis. MZ, NMF, LPB, JGS, AKP, BAM, LCO, JLG, and VR designed and coordinated the study. MZ, VR, and JLG wrote the manuscript.

## Supplementary Material

Supplemental data

Supplemental data set 1

## Version 1 05/10/2022

In-Press Preview

## Version 2 06/22/2022

Electronic publication

## Version 3 06/05/2024

Replaced missing paragraph from Results section
