# Age-dependent gray matter demyelination is associated with leptomeningeal neutrophil accumulation

**DOI:** 10.1172/jci.insight.183445

**Published:** 2024-06-24

**Authors:** Michelle Zuo, Naomi M. Fettig, Louis-Philippe Bernier, Elisabeth Pössnecker, Shoshana Spring, Annie Pu, Xianjie I. Ma, Dennis S.W. Lee, Lesley A. Ward, Anshu Sharma, Jens Kuhle, John G. Sled, Anne-Katrin Pröbstel, Brian A. MacVicar, Lisa C. Osborne, Jennifer L. Gommerman, Valeria Ramaglia

Original citation *JCI Insight*. 2022;7(12):e158144. https://doi.org/10.1172/jci.insight.158144

Citation for this erratum: *JCI Insight*. 2024;9(12):e183445. https://doi.org/10.1172/jci.insight.183445

During the final production of this manuscript, a paragraph was inadvertently deleted from the Results section. The missing paragraph is provided below. The HTML and PDF files have been updated online.

A key hallmark of progressive MS is a reduction in brain volume driven in part by atrophy in the cortical gray matter (27, 28). To test if the SJL/J A/T EAE model in old mice exhibits brain atrophy, we followed old and young SJL/J mice for 40 days post-A/T and assessed brain volume by T2-weighted 7-Tesla magnetic resonance imaging (MRI) at 2 time points — acute (day 11) and postacute (day 40) (Figure 4F). Normalizing to skull length measurements taken from the nasal cavity to the base of the skull (the axial MRI view — see Figure 4G), we found that old SJL/J A/T EAE mice exhibited lower brain volume compared with age- and sex-matched naive controls at the postacute time point, while young EAE mice exhibited similar brain volume compared to their appropriate controls (Figure 4H). We observed similar differences when normalizing to body weight or length of the entire skull (data not shown). Moreover, when expressed as a percentage change from the mean brain volume of age-matched controls, old but not young SJL/J A/T EAE mice at the postacute stage exhibited a significant decrease from controls (Figure 4I). Last, a strong negative correlation was observed between brain volume and EAE severity (Figure 4J). We also noted a significant decrease in somatosensory cortex volume between old SJL/J A/T EAE mice and their age-matched controls at the postacute time point (Figure 4K). These data suggest that severe, prolonged EAE results in diminished brain volume in old SJL/J A/T EAE mice, detectable by MRI as early as day 40 post-A/T.

*JCI Insight* regrets the error.

## Version 1 06/24/2024

Electronic publication

