# Supplementary material for: Age-dependent gray matter demyelination is associated with leptomeningeal neutrophil accumulation
Source: JCI Insight. 2022 May 10;7(12):e158144. doi: 10.1172/jci.insight.158144 (PMC9309059; doi:10.1172/jci.insight.158144)
Supplement: Supplemental data [file jciinsight-7-158144-s008.pdf]

*Age-dependent grey matter demyelination is associated with leptomeningeal neutrophil accumulation*

Michelle Zuo[1], Naomi Fettig[2], Louis-Philippe Bernier[3], Elisabeth Pössnecker[4], Shoshana Spring[5], Annie Pu[1], Xianjie I. Ma[1], Dennis S. W. Lee[1], Lesley Ward[1], Anshu Sharma[1], Jens Kuhle[4], John G. Sled[5,6], Anne-Katrin Pröbstel[4], Brian MacVicar[3], Lisa Osborne[2], Jennifer L. Gommerman[1]*, and Valeria Ramaglia[1]*

[1]Department of Immunology, University of Toronto, Toronto, Ontario, Canada.

[2]Department of Microbiology and Immunology, Life Sciences Institute, University of British Columbia, Vancouver, BC, Canada.

[3]Department of Psychiatry, University of British Columbia, Vancouver, BC, Canada.

[4]Neurologic Clinic and Policlinic & Research Center for Clinical Neuroimmunology and Neuroscience Basel, Departments of Head, Spine and Neuromedicine, Biomedicine, and Clinical Research, University Hospital and University of Basel, Basel, Switzerland.

[5]Mouse Imaging Centre, Hospital for Sick Children, Toronto, Ontario, Canada.

[6]Department of Medical Biophysics, University of Toronto, Toronto, Ontario, Canada.

*Corresponding authors, jen.gommerman@utoronto.ca; v.ramaglia@utoronto.ca

*Mailing addresses:

Jennifer Gommerman
Department of Immunology, Temerty Faculty of Medicine
University of Toronto
1 King's College Circle, Rm 7233
Toronto, ON, M5S 1A8, Canada
Phone: (416) 978-6959

Valeria Ramaglia
Department of Immunology, Temerty Faculty of Medicine
University of Toronto
1 King's College Circle, Rm 7233
Toronto, ON, M5S 1A8, Canada
Phone: (416) 978-6959

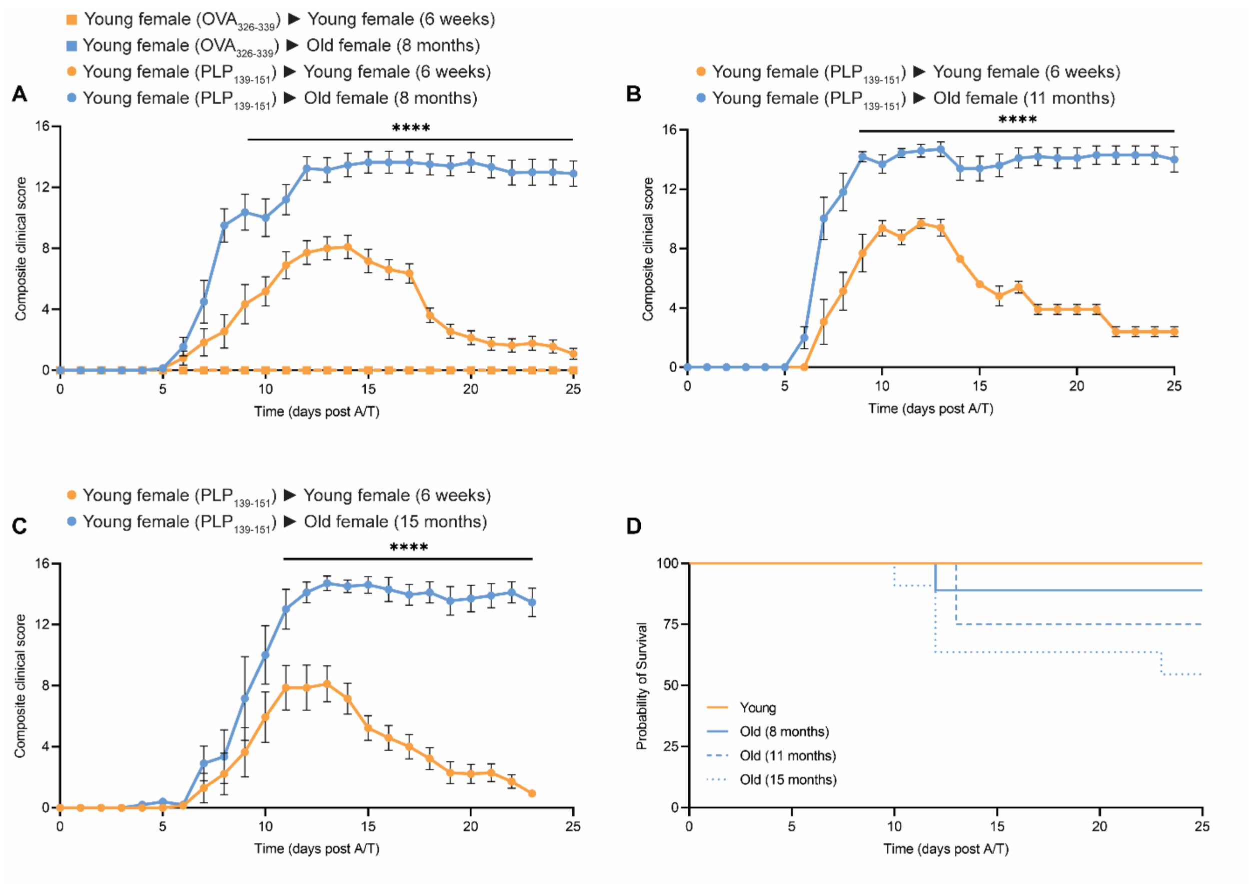


**Supplemental Figure 1. The non-remitting phenotype of SJL/J A/T EAE is reproducible.** (**A**) Clinical course of A/T EAE using cells from $PLP_{139\text{-}151}$- or $OVA_{326\text{-}339}$-primed donors. Only mice receiving cells from $PLP_{139\text{-}151}$-primed donors developed clinical disease (young n=18, old n=11), while mice receiving cells from $OVA_{326\text{-}339}$-primed donors remained asymptomatic (young, n=4; old, n=3). (**B**) Clinical course of A/T EAE in 6 -week (n=8) vs 11-month-old (n=8) recipients. (**C**) Clinical course of A/T EAE in 6-week (n=8) vs 15-month-old (n=8) recipients. (**D**) Kaplan-Meier curve showing age-dependent survival in SJL/J A/T EAE mice. **A, B, C** = Stats by two-way ANOVA with Bonferroni correction for multiple comparisons, error bars indicate mean ± SEM. Experiments were all performed at U of T. ****$P \leq 0.0001$.

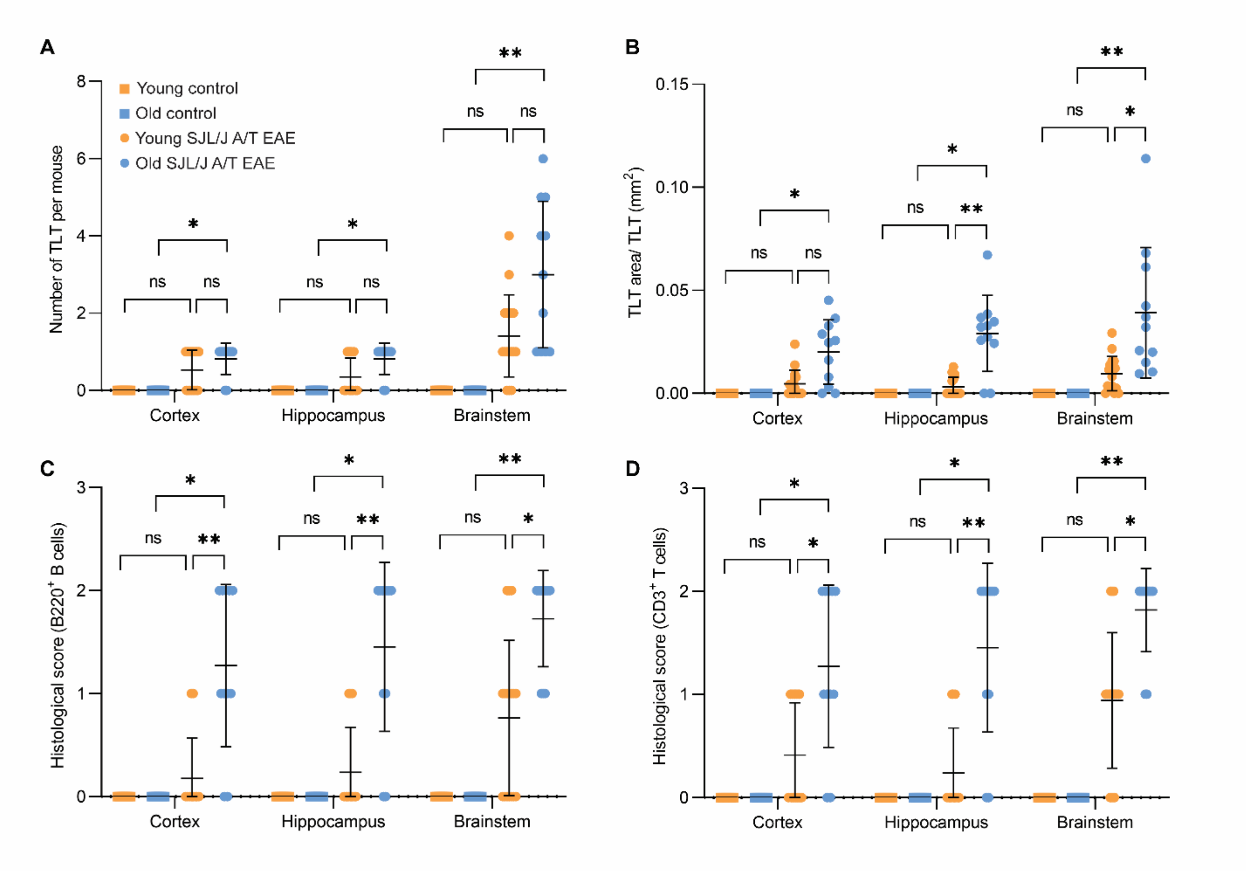


**Supplemental Figure 2. Ageing induces accumulation of lymphocytes in leptomeninges adjacent to subpial and periventricular areas in SJL/J A/T EAE mice at post-acute disease stage.** (**A**) Number and (**B**) area of TLT in young (n=17) and old (n=11) SJL/J A/T EAE mice at post-acute stage compared to unimmunized, age-matched controls. Histological score indicating presence of (**C**) $B220^+$ B cells and (**D**) $CD3^+$ T cells in the TLTs at subpial and periventricular areas. **A-D** = Stats by two-way ANOVA with Tukey's correction for multiple comparisons, error bars indicate mean ± SD. *$p \leq 0.05$, **$p \leq 0.01$.

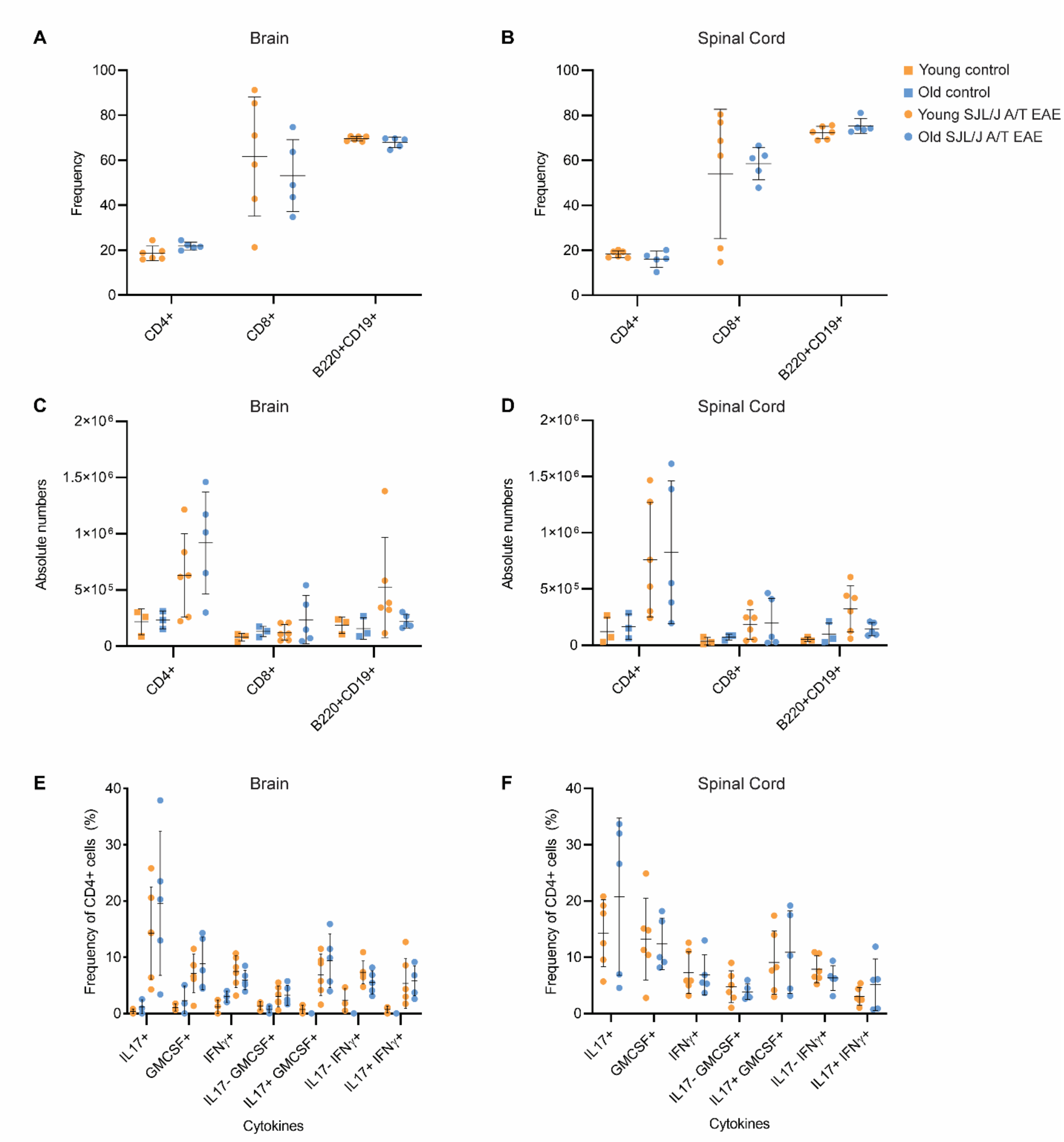


**Supplemental Figure 3. Ageing does not impact the frequency of lymphocytes or the ability of T cells to produce cytokines in SJL/J A/T EAE mice at peak disease stage.** Whole brains and spinal cords of young (n=6) and old (n=5) SJL/J A/T EAE and naïve (brain, n=3 per age) mice were analyzed by flow cytometry. (**A-B**) Frequencies of B220$^+$CD19$^+$ B cells (of CD45$^+$ single cells), CD4$^+$ and CD8$^+$ T cells (of CD3$^+$CD45$^+$ single cells) in the brains and spinal cords of old vs young SJL/J A/T EAE mice. Stats by Mann-Whitney U showed no statistical difference between the groups. Refer to methods for full T cell stimulation protocol. (**C-D**) Absolute numbers of B220$^+$CD19$^+$ B cells (of CD45$^+$ single cells), CD4$^+$ and

$CD8^+$ T cells (of $CD3^+CD45^+$ single cells) in the brains and spinal cords of old vs young SJL/J A/T EAE mice compared to age-matched, unimmunized controls. Stats by one-way ANOVA showed no statistical difference between groups. (**E-F**) Frequencies of cytokine-producing $CD4^+$ T cells was assessed using intra-cellular staining for GM-CSF, IFNγ, and IL-17. Statistical test by Mann-Whitney showed no significant differences between the groups, error bars indicate mean ± SD.

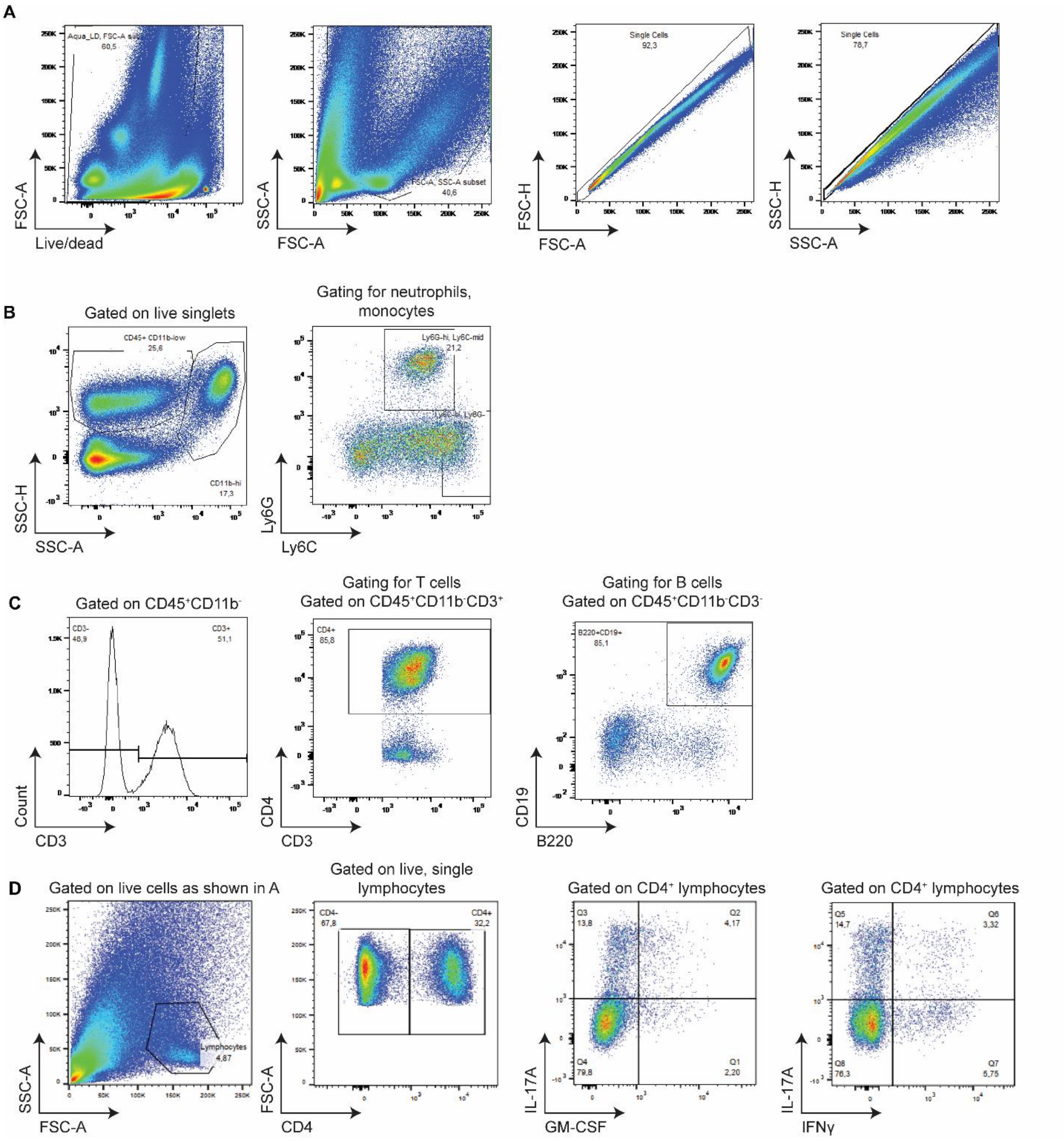


**Supplemental Figure 4. Gating strategies for identification of T cells, B cells, neutrophils, and cytokine-producing cells. (A)** Pre-gating for live, single cells. **(B)** Gating for identification of neutrophils ($CD45^+CD11b^+Ly6G^+Ly6C^-$mid). **(C)** Gating for identification of T cells ($CD45^+CD11b^-CD3^+CD4^+$) and B cells ($CD45^+CD11b^-CD3^-B220^+CD19^+$). **(D)** Pre-gating for lymphocytes, following by identification of $CD4^+$ cytokine producing cells.

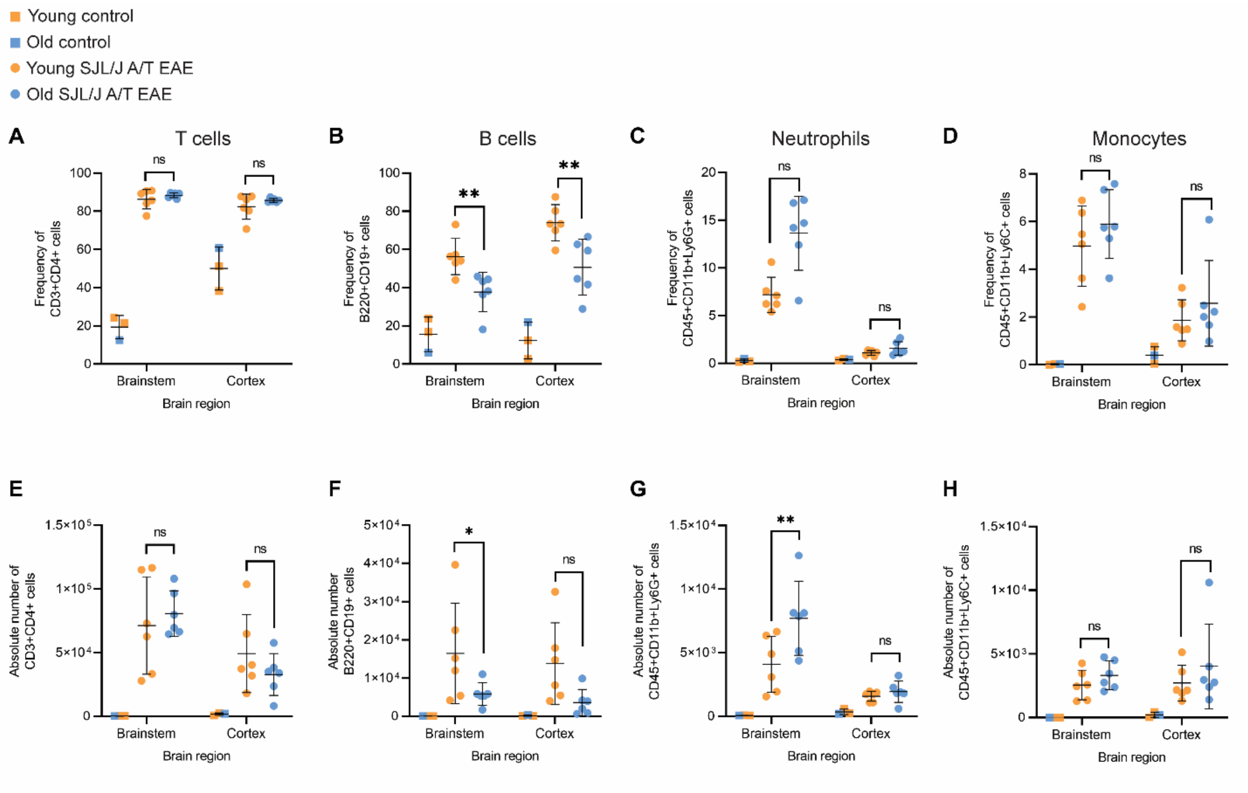


**Supplemental Figure 5. Flow cytometry of cortex and brainstem from old vs young SJL/J A/T EAE mice reveals differences in B cell composition only.** Brainstem and cortex were separately dissected from old (n=6) vs young (n=6) SJL/J A/T EAE mice at the acute timepoint and subjected to analysis by flow cytometry. Controls represented by age-matched unimmunized SJL/J mice (old, n=1, young, n=2). Despite no change in **(A, E)** T cell, **(C, G)** neutrophils, or **(D, H)** monocytes in number or frequency, we observed a decrease in density of **(B, F)** B cells between old and young SJL/J A/T EAE mice. Stats by one-way ANOVA (absolute number) or Kruskal-Wallis (frequency) with correction for multiple comparisons, error bars indicate mean ± SD. *$p \leq 0.05$, **$p \leq 0.01$.

Supplemental Tables

**Supplemental table 1. Antibodies used for immunohistochemistry, immunofluorescence, and flow cytometry.**

| **Primary Antibody** | ***Clone*** | **Target** | **Dilution** | **Antigen Retrieval** | **Source** |
|---|---|---|---|---|---|
| *Immunohistochemistry* | | | | | |
| PLP | mAb, plpc1 | Proteolipid protein (Myelin) | 1:100 | Tris-EDTA | Bio-Rad, MCA839G |
| Iba-1 | mAb, EPR16589 | Ionized alcium binding adaptor molecule 1 (microglia/macrophages) | 1:4000 | Tris-EDTA | Abcam, ab92305 |
| GFAP | pAb | Glial fibrillary acidic protein (astrocytes) | 1:4000 | Tris-EDTA | Dako, Z0334 |
| Synaptophysin | mAb, SY38 | Synapses | 1:2500 | Tris-EDTA | ThermoFisher, MA1-213 |
| Pan-Neurofilament | pAb | Axons | 1:100 | Tris-EDTA | Abcam, ab204893 |
| Neurofilament light chain | mAb, 8A1 | Axons | 1:100 | Tris-EDTA | Santa Cruz Biotech, sc-20012 |
| CD3 | mAb, SP7 | T cells | 1:100 | Citrate | Abcam, ab16669 |
| CD20 | mAb, L26 | B cells | 1:100 | Citrate | Abcam, ab9475 |
| *Immunofluorescence* | | | | | |
| CD45-AF594* | mAb, 30-F11 | Lymphocytes and myeloid cells | 1:200 | - | Biolegend, 103144 |
| CD3-AF594* | mAb, 17A2 | T cells | 1:100 | - | Biolegend, 100240 |
| B220-AF488* | mAb, RA3-6B2 | B cells | 1:100 | - | eBioscience 53-0452-82 |
| Ly6G-PE* | mAb, 1A8 | Neutrophils | 1:100 | - | eBioscience, 12-9668-82 |
| Fibronectin* | mAb, GW20021F | Extracellular matrix | 1:100 | - | Sigma, 53-0452-83 |
| *Flow cytometry* | | | | | |

| | | | | | |
|---|---|---|---|---|---|
| CD45.1-BUV395 | mAb, A20 | Lymphocytes and myeloid cells | 1:100 | - | BD Horizon, 565212 |
| CD19-eF450 | mAb, 1D3 | B cells | 1:200 | - | eBioscience, 48-0193-82 |
| B220-BV605 | mAb, RA3-6B2 | B cells | 1:200 | - | Biolegend, 103244 |
| CD3-BV711 | mAb, 17A2 | T cells | 1:200 | - | Biolegend, 100241 |
| Ly6C-PerCP-Cy5.5 | mAb, HK1.4 | Monocytes | 1:200 | - | eBioscience, 45-5932-82 |
| Ly6G-PE | mAb, 1A8 | Neutrophils | 1:200 | - | eBioscience, 12-9668-82 |
| CD11b-PE-Cy7 | mAb, M1/70 | Myeloid cells | 1:200 | - | eBioscience, 25-0112-82 |
| CD4-APC | mAb, RM4-5 | MHC class II restricted T cells | 1:200 | - | eBioscience, 17-0042-82 |
| CD8α-PE-Cy7 | mAb, 53-6.7 | MHC class I restricted T cells | 1:200 | - | eBioscience, 25-0081-82 |
| CD11c-APC-Cy7 | mAb, N418 | Myeloid cells | 1:200 | - | Biolegend, 117324 |
| GM-CSF-FITC | mAb, MP1-22E9 | Granulocyte-macrophage colony-stimulating factor | 1:100 | - | eBioscience, 11-7331 |
| IL17a-PerCP-Cy5.5 | mAb, 17B7 | Interleukin 17 alpha | 1:100 | - | eBioscience, 45-7177 |
| IFNγ-PE-Cy7 | mAb, XMG1.2 | Interferon gamma | 1:200 | - | eBioscience, 25-7311 |
| Brefeldin A | | | 1:1000 | - | eBioscience, 00-4506-51 |
| Aqua fluorescein viability dye | | | 1:1000 | - | ThermoFisher, L34965 |
| mAb, monoclonal antibody; pAb, polyclonal antibody; Tris-EDTA, 10mM Tris 1mM EDTA buffer pH 9.0; Citrate, 10mM citrate buffer pH 6.0; the asterisk indicates antibodies used on frozen sections | | | | | |

**Supplementary Table 2. Donor demographics**

| Case | Sex | PMD (h:min) | Type of MS | DD (years) | COD |
|---|---|---|---|---|---|
| 1 | F | 08:40 | SPMS | 26 | Respiratory insufficiency to (uro)sepsis |
| 2 | F | 10:40 | SPMS | 29 | Euthanasia |
| 3 | F | 07:30 | SPMS | 34 | Euthanasia |
| 4 | F | 11:50 | PPMS | 22 | Respiratory failure with end stage MS |
| 5 | M | 08:15 | SPMS | 30 | Pneumonia, cachexia and dehydration |
| 6 | F | 09:05 | SPMS | 18 | Euthanasia |
| 7 | F | 08:35 | PPMS | 29 | Aspiration pneumonia |
| 8 | F | 05:45 | PPMS | 22 | Sepsis |
| 9 | F | 08:25 | SPMS | 11 | Natural death |
| 10 | M | 11:00 | SPMS | >12 | Exact cause unknown, infection 2 days prior to death |
| 11 | F | 08:40 | PPMS | 29 | Euthanasia |
| 12 | F | 08:25 | SPMS | 34 | Respiratory insufficiency secondary to pneumonia |
| 13 | M | 07:30 | - | - | - |
| 14 | F | 06:45 | PPMS | 29 | Cardiac asthma |
| 15 | F | 07:30 | SPMS | 39 | Bronchitis/ aspiration pneumonia |
| 16 | M | 10:45 | RRMS | 22 | Euthanasia |
| 17 | F | 08:00 | SPMS | 42 | Pneumonia |
| 18 | M | 06:20 | PPMS | 32 | Respiratory insufficiency |
| 19 | F | 10:05 | SPMS | 26 | Cardiovascular event and dehydration |
| 20 | F | 07:50 | SPMS | 50 | Euthanasia |
| 21 | M | 10:45 | RRMS | 22 | Euthanasia |
| 22 | F | 07:05 | SPMS | 34 | Cachexia with slowly progressive MS and metastatic breast cancer |
| 23 | F | 09:35 | PPMS | 22 | Cardiac asthma |
| 24 | F | 04:35 | SPMS | 34 | Aspiration pneumonia |
| 25 | M | 09:15 | - | - | - |
| 26 | M | 07:30 | PPMS | 32 | Respiratory failure due to pneumonia |
| 27 | F | 09:45 | SPMS | 35 | Euthanasia |
